# Supplementary material for: Midlife socioeconomic position and old-age dementia mortality: a large prospective register-based study from Finland
Source: BMJ Open. 2020 Jan 6;10(1):e033234. doi: 10.1136/bmjopen-2019-033234 (PMC6955538; doi:10.1136/bmjopen-2019-033234)
Supplement: Supplementary data [file bmjopen-2019-033234supp001.pdf]

Supplementary Table 1. Classification of chronic health conditions used as covariates in the study

Condition	Hospital diagnoses (ICD-10)	Prescription medication (ATC)	Special reimbursement category (Finnish disease code)
Alcohol-related diseases and accidental poisoning by alcohol	F10–19, G31.2, G40.51, G62.1, G72.1, I42.6, K29.2, K70, K86.0, O35.4, X45		
Asthma and other COPD	J43–46		203
Diabetes	E10–14	A10	103
Heart disease	I00–09, I20–52		201, 206, 207

Abbreviations: ATC, Anatomical Therapeutic Chemical; COPD, chronic obstructive pulmonary diseases; ICD, International Classification of Diseases
