# Supplementary material for: Midlife socioeconomic position and old-age dementia mortality: a large prospective register-based study from Finland
Source: BMJ Open. 2020 Jan 6;10(1):e033234. doi: 10.1136/bmjopen-2019-033234 (PMC6955538; doi:10.1136/bmjopen-2019-033234)
Supplement: Supplementary data [file bmjopen-2019-033234supp002.pdf]

Supplementary Table 2. Kaplan-Meier survival probabilities at specific ages by midlife a) education, b) occupational social class and c) household income quintile (Q1=highest, Q5=lowest), Finnish men and women in 2001–2016

		Dementia		Other causes	
	Age	Survivor function	95% CI	Survivor function	95% CI
a) Education					
Tertiary	70	1.00		1.00	
	75	0.99	0.99–1.00	0.91	0.89–0.92
	80	0.97	0.97–0.98	0.80	0.78–0.82
	85	0.92	0.90–0.92	0.63	0.61–0.65
	90	0.76	0.75–0.78	0.43	0.41–0.44
	95	0.53	0.50–0.56	0.23	0.22–0.25
	100	0.29	0.24–0.35	0.09	0.07–0.11
Basic	70	1.00		1.00	
	75	0.99	0.99–1.00	0.88	0.87–0.89
	80	0.97	0.97–0.97	0.74	0.73–0.74
	85	0.89	0.89–0.90	0.56	0.55–0.56
	90	0.73	0.73–0.74	0.36	0.36–0.37
	95	0.48	0.47–0.49	0.19	0.18–0.20
	100	0.22	0.20–0.24	0.06	0.05–0.07
b) Occupational social class					
Non-manual	70	1.00		1.00	
	75	1.00	0.99–1.00	0.92	0.90–0.93
	80	0.97	0.97–0.98	0.80	0.79–0.81
	85	0.91	0.90–0.92	0.64	0.63–0.65
	90	0.76	0.75–0.77	0.44	0.43–0.45
	95	0.51	0.49–0.52	0.25	0.24–0.26
	100	0.24	0.21–0.27	0.10	0.09–0.11
Manual	70	1.00		1.00	
	75	0.99	0.99–0.99	0.87	0.85–0.88
	80	0.97	0.96–0.97	0.71	0.70–0.72
	85	0.89	0.89–0.90	0.53	0.52–0.54
	90	0.73	0.72–0.73	0.34	0.34–0.35
	95	0.46	0.45–0.47	0.17	0.17–0.18
	100	0.21	0.18–0.24	0.06	0.05–0.07

	Age	Dementia		Other causes	
		Survivor function	95% CI	Survivor function	95% CI
c) Household income					
Q1	70	1.00		1.00	
	75	0.99	0.99–1.00	0.91	0.90–0.92
	80	0.97	0.97–0.98	0.79	0.78–0.80
	85	0.92	0.91–0.92	0.62	0.61–0.63
	90	0.75	0.74–0.76	0.43	0.42–0.44
	95	0.51	0.49–0.53	0.24	0.22–0.25
	100	0.24	0.20–0.28	0.09	0.08–0.11
Q5	70	1.00		1.00	
	75	0.99	0.99–1.00	0.85	0.83–0.87
	80	0.96	0.95–0.97	0.68	0.67–0.70
	85	0.88	0.87–0.89	0.50	0.48–0.51
	90	0.71	0.70–0.72	0.31	0.30–0.32
	95	0.45	0.43–0.47	0.15	0.14–0.16
	100	0.23	0.19–0.26	0.04	0.04–0.06
