# Supplementary material for: Midlife socioeconomic position and old-age dementia mortality: a large prospective register-based study from Finland
Source: BMJ Open. 2020 Jan 6;10(1):e033234. doi: 10.1136/bmjopen-2019-033234 (PMC6955538; doi:10.1136/bmjopen-2019-033234)
Supplement: Author's manuscript [file bmjopen-2019-033234.draft_revisions.pdf]

BMJ Open

BMJ Open is committed to open peer review. As part of this commitment we make the peer review history of every article we publish publicly available.

When an article is published we post the peer reviewers' comments and the authors' responses online. We also post the versions of the paper that were used during peer review. These are the versions that the peer review comments apply to.

The versions of the paper that follow are the versions that were submitted during the peer review process. They are not the versions of record or the final published versions. They should not be cited or distributed as the published version of this manuscript.

BMJ Open is an open access journal and the full, final, typeset and author-corrected version of record of the manuscript is available on our site with no access controls, subscription charges or pay-per-view fees (http://bmjopen.bmj.com).

If you have any questions on BMJ Open's open peer review process please email info.bmjopen@bmj.com

BMJ Open

Midlife socioeconomic position and old-age dementia mortality: a large prospective register-based study from Finland

Journal:	*BMJ Open*
Manuscript ID	bmjopen-2019-033234
Article Type:	Research
Date Submitted by the Author:	26-Jul-2019

Complete List of Authors:	Korhonen, Kaarina; University of Helsinki, Population Research Unit<br>Einiö, Elina; University of Helsinki<br>Leinonen, Taina; Finnish Institute of Occupational Health,<br>Tarkiainen , Lasse ; University of Helsinki<br>Martikainen, Pekka; University of Helsinki

Keywords:	Dementia < NEUROLOGY, Socioeconomic factors, Alzheimer's disease, Register study

Note: The following files were submitted by the author for peer review, but cannot be converted to PDF. You must view these files (e.g. movies) online.
Image1.tif

SCHOLARONE™
Manuscripts

Midlife socioeconomic position and old-age dementia mortality: a large prospective register-based study from Finland

Kaarina Korhonen[1], Elina Einiö[1,2,3], Taina Leinonen[4], Lasse Tarkiainen[1], Pekka Martikainen[1, 3, 5]

[1] Population Research Unit, Faculty of Social Sciences, University of Helsinki, Helsinki, Finland

[2] Department of Social Policy, London School of Economics and Political Science, London, United Kingdom

[3] Max Planck Institute for Demographic Research, Rostock, Germany

[4] Finnish Institute of Occupational Health, Helsinki, Finland

[5] Department of Public Health Sciences, Stockholm University, Stockholm, Sweden

Correspondence to:

Kaarina Korhonen

Faculty of Social Sciences

P.O. Box 18, 00014 University of Helsinki, Finland

+358 50 3199 388

kaarina.korhonen@helsinki.fi

Word count: 3 476

For peer review only

Abstract

Objectives To assess the association between multiple indicators of socioeconomic position and dementia-related death, and to estimate the contribution of dementia to socioeconomic differences in overall mortality at older ages.

Design Prospective population-based register study.

Setting Finland.

Participants 11% random sample of men and women aged 70–87 resident in Finland at the end of 2000 (N=54 964).

Main outcome measure Incidence rates, Kaplan-Meier survival probabilities and Cox regression hazard ratios of dementia mortality in 2001–2016 by midlife education, occupational social class and household income measured at ages 53–57 years.

Results During the 528 387 person-years at risk, 11 395 individuals died from dementia (215.7 per 10 000 person-years). Lower midlife education, occupational social class and household income were associated with higher dementia mortality, and the differences persisted to the oldest old ages. Compared to mortality from all other causes, however, the socioeconomic differences emerged later and were altogether smaller. Dementia accounted for 30% of the difference between low and high education groups in overall mortality at age 70+, and for 25% of the difference between lowest and highest household income quintiles. All indicators of socioeconomic position were independently associated with dementia mortality, low household income being the strongest independent predictor (HR=1.24, 95% confidence interval 1.16–1.32), followed by basic education (HR=1.14, 1.06–1.23). Manual occupational social class was related to a 6% higher hazard (HR=1.06, 1.01–1.11) compared to white-collar social class. Adjustment for midlife economic activity, baseline marital status and chronic health conditions attenuated the excess hazard of low midlife household income, although significant effects remained.

Conclusion .

For peer review only - http://bmjopen.bmj.com/site/about/guidelines.xhtml

Several indicators of socioeconomic position predict dementia mortality independently and these differences persist into the oldest old ages. The results demonstrate that dementia is among the most important contributors to socioeconomic inequalities in overall mortality at older ages.

Strengths and limitations of this study

- We used longitudinal registry data that permits a 15-year follow-up of dementia mortality with no attrition or recall bias.
- All indicators of socioeconomic position were measured in midlife in order to avoid selection to socioeconomic groups on the basis of cognitive decline.
- This is the first study to show the contribution of dementia to the socioeconomic inequalities in overall mortality at older ages.
- We used individual death records and could only identify dementia cases that were recorded on the death certificate.

For peer review only

For peer review only - http://bmjopen.bmj.com/site/about/guidelines.xhtml

Introduction

Socioeconomic inequality in health and mortality is one of the most consistent findings in the demographic and social epidemiological literature. Lower education, occupational social class and income are strong predictors of all-cause and cause-specific mortality particularly among the working-age population, but inequalities are clear also at older ages (1–4). Among the ageing population, the key factors affecting morbidity and disability are Alzheimer's disease and other forms of progressive dementia. Globally, an estimated 47 million people lived with dementia in 2015, and the number is projected to triple by 2050 (5). In England and Wales, dementia has already become the leading cause of death (6). Despite the growing societal impact, however, no comprehensive understanding exists about the socioeconomic patterns of dementia mortality.

Educational inequalities in dementia mortality have previously been reported in studies following individuals from midlife or younger old ages (7,8) but also at the oldest ages (8,9). In a Norwegian health examination study, an educational pattern was present only among cohorts aged below 70 at baseline but not among those aged 70 and over. Similarly, among a Finnish cohort aged 90 and over, no statistically significant educational gradient in dementia mortality emerged (9). The lack of educational pattern among the oldest old may relate selective survival, indicating that people surviving to this age is more homogeneous in terms of health-related characteristics. However, the finding may also relate to the fact that the oldest cohorts are relatively homogeneous in terms of education and, thus, other indicators of socioeconomic position may be more suitable in identifying disadvantaged population subgroups among these cohorts (2,10). Moreover, previous studies suggest that among adults in general, overall mortality disparities are greater or have increased to a greater extent in terms of occupational social class (11) and income (12,13) than education. Among the Finnish cohort of nonagenarians (9), occupational social class was a strong predictor of dementia mortality with a 3-fold hazard of dementia death among the unskilled manual workers compared to upper non-manuals. Personal income in midlife was not related to dementia mortality among a cohort of Norwegian men (14). To our knowledge, no previous study has assessed

For peer review only - http://bmjopen.bmj.com/site/about/guidelines.xhtml

inequalities in dementia mortality by household income, a socioeconomic indicator that is more directly related to material resources available to the individual and that more rigorously captures the living conditions of the most disadvantaged population subgroups.

This study contributes to the existing knowledge by assessing socioeconomic inequalities in dementia mortality using multiple indicators of socioeconomic position, including education, occupational social class and household income. More specifically, the aims of the study were to 1) investigate the magnitude of socioeconomic inequalities in dementia mortality in relation to age, and compare the patterns to those in mortality from all other causes of death, 2) to assess the contribution of dementia to the socioeconomic inequalities in overall mortality at older ages, and 3) to assess whether education, occupational social class and household income are independently related to dementia mortality once the other indicators are taken into account. This was because different indicators of socioeconomic position are correlated but each of them may have independent associations with mortality. In order to capture potential mediating pathways, we estimated models adjusted for marital status and various chronic health conditions. We used longitudinal registry data on a large population-based sample, which permits a 15-year follow-up of dementia-related deaths with no attrition or recall bias. All indicators of socioeconomic position were measured in midlife in order to avoid selection to socioeconomic groups on the basis of cognitive decline.

Methods

Sample

We used an 11% random sample of the Finnish population in 1987–2007 drawn from the Statistics Finland population register, which covers all permanent residents. Statistics Finland linked the sample with information from various administrative registers including the national Death Register and healthcare registers using unique personal identification numbers assigned to all permanent residents.

In the present study, we included men and women aged 70–87 at the end of 2000. For these cohorts, midlife socioeconomic characteristics could be identified using information from the Population Censuses

For peer review only - http://bmjopen.bmj.com/site/about/guidelines.xhtml

conducted in 1970, 1975, 1980 and 1985. Individuals with missing census information due to residing outside of Finland (n=920) and those with missing household income information due to institutional residence (n=401) were excluded. 7 individuals emigrated during the first year of follow-up and thus were excluded from the analyses. The analytic sample consisted of 54,964 individuals.

Mortality data

Dates and causes of death were obtained from the Death Register. Dementia-related deaths were identified using the International Classification of Diseases 10th revision (ICD-10) codes F00–03 and G30 as the underlying or any of the three contributory causes of death reported on the death certificate. We identified 11,395 persons who died from dementia and 30,637 persons who died from other causes during the follow-up in 2001–2016.

Indicators of socioeconomic position

The information of all indicators of socioeconomic position was derived from the quinquennial population censuses of 1970–1985. A particular census year was chosen on the basis of the study subject's age so that the indicators were measured at around the age of 55 (range 53–57) for all. Education was indicated as the highest achieved qualification, categorised as tertiary (generally 13+ years of education; International Standard Classification of Education ISCED-1997 codes 5–6), secondary (10–12 years, ISCED 3–4), and basic education (9 years, ISCED 0–2) or education unknown. Occupational social class comprised five groups, classified as white collar, manual, self-employed farmer, other self-employed, and unknown. For non-employed individuals in the census year, we tracked information from previous years. Household income indicated the taxable annual income of all household members. This includes all income received in money or monetary benefit subject to tax, therefore excluding tax-free income transfers such as child benefit, housing allowance and social assistance. The information was obtained from the Finnish Tax Administration and the Social Insurance Institution of Finland. We adjusted for household composition

For peer review only - http://bmjopen.bmj.com/site/about/guidelines.xhtml

using the OECD-modified equivalence scale (15). Income quintiles were formed based on the household income distribution in the population census year.

Covariates

The analyses incorporated information of economic activity measured from the census year because being out of the labour market may indicate poor health and affect dementia risk independently but also lead to reduced household income. Economic activity was classified as being in the labour force, retired and other inactive. Marital status was measured at baseline (the end of 2000), classified as married, divorced, widowed and never married. Baseline chronic health conditions included indicators of vascular and lifestyle risk factors for dementia (16), and were identified from health registers in the five-year period before the baseline, covering 1996–2000. We used the diagnostic records of the hospital discharge register and patient censuses of the National Institute for Health and Welfare, and the records of prescription medicine purchases and of entitlement to special reimbursement for the medication expenses for certain chronic diseases maintained by the Social Insurance Institution of Finland. We included indicators for alcohol-related diseases and accidental poisoning by alcohol (ICD-10 codes F10–19, G31.2, G40.51, G62.1, G72.1, I42.6, K29.2, K70, K86.0, O35.4, X45), asthma and other chronic obstructive pulmonary disease (COPD) (ICD-10 codes J43–46, Finnish disease category code 203), diabetes (ICD-10 codes E10–14, Anatomical Therapeutic Chemical (ATC) codes A10, Finnish disease category code 103), heart disease (ICD-10 codes I00–09 and I20–52, Finnish disease category codes 201, 206 and 207) and stroke (ICD-10 codes I60–66 and G45). To account for potential regional variance in socioeconomic characteristics and mortality, we included dummies for region of residence (Western Finland, Helsinki capital region, rest of Southern Finland, Eastern Finland, and Lapland). The variable accounting for the degree of urbanisation of the municipality of residence was based on the proportion of population living in urban settlements and the population of the largest urban settlement in the municipality (urban, semi-urban and rural).

For peer review only - http://bmjopen.bmj.com/site/about/guidelines.xhtml

Statistical analyses

We followed the study population for dementia mortality from 1 January 2001 until 31 December 2016. Individuals were censored on the date of death, the end of the year preceding emigration, or at the end of 2016, whichever came first.

For descriptive statistics, we calculated age-adjusted dementia mortality rates per 10,000 person-years by indicators of socioeconomic position, economic activity, marital status and chronic health conditions. In order to assess the magnitude of socioeconomic inequalities in relation to age, we estimated Kaplan–Meier survival functions by education, occupational social class and household income. In these analyses, we contrasted the survival functions of the highest and lowest education groups, white-collar employees and manual workers and the highest and lowest household income quintiles. The equality of survival functions was tested using log-rank tests. For the comparison between dementia mortality and the more general mortality patterns, separate Kaplan-Meier survival functions were estimated for mortality from all other causes of death.

To assess the contribution of dementia mortality to socioeconomic differences in overall mortality, we calculated absolute rate differences in dementia and total mortality between socioeconomic groups (basic vs. tertiary education, manual vs. white-collar occupational social class, lowest vs. highest household income quintile). The contribution was determined by the rate difference in dementia mortality as a percentage of the rate difference in total mortality. The rate differences by indicators of socioeconomic position and age are shown in Supplementary Table 1.

To estimate the independent associations between each indicator of socioeconomic position and dementia mortality, we used Cox regression models. Attained age in years was used as the time scale, and thus all analyses adjusted for the confounding effect of age (17). We first estimated crude associations between each indicator and dementia mortality, adjusting for calendar year dummies, gender, region of residence and the degree of urbanisation (model 1). Model 2 included education, occupational social class and household

For peer review only - http://bmjopen.bmj.com/site/about/guidelines.xhtml

income as covariates, thus showing mutually adjusted associations. Midlife economic activity was adjusted for in model 3. We further adjusted for baseline marital status and chronic health conditions in model 4 to assess the extent to which these factors attenuated the relative hazard attached to each socioeconomic indicator.

We tested for interactions between gender and each socioeconomic indicator using likelihood ratio test. Interactions were statistically nonsignificant (p>0.05), and thus we conducted all analyses for men and women combined. We also tested for interactions of all pairwise combinations of the socioeconomic indicators, adjusting for the covariates of model 1. These interactions were all statistically non-significant (p>0.05). All analyses were performed using Stata 15.1 (18).

Patient and public involvement

No patients were involved in setting the research question or the outcome measures, nor were they involved in developing plans for design or implementation of the study. No patients were asked to advise on interpretation or writing up of results. There are no plans to disseminate the results of the research to study participants or the relevant patient community.

Results

Table 1 shows the distribution of the study population by indicators of midlife socioeconomic position, economic activity and baseline characteristics. The vast majority of individuals (77.2%) had no higher than basic education, and manual employees formed the largest occupational social class (43.6%). Higher household income quintiles were over-represented among the study population due to greater mortality of the lower income groups between the time of measurement of midlife income and the baseline. During the 528 387 person-years at risk 11 395 individuals died from dementia, the average age-adjusted dementia mortality rate being 191.2 and 228.1 per 10,000 person-years among men and women, respectively. The rate was higher for those with lower education, occupational social class and household income, and also for the non-married and people with chronic health conditions apart from asthma and other COPD.

For peer review only - http://bmjopen.bmj.com/site/about/guidelines.xhtml

[Insert Table 1 about here]

Kaplan–Meier survival functions in Figure 1 show that dementia mortality differed by all indicators of socioeconomic position (log rank test, $p<0.001$ for each indicator), and that the age patterns differed between the indicators. The inequalities emerged at an earlier age when socioeconomic position was measured in terms of household income (Panel C) compared to education (Panel A) and occupational social class (Panel B). At the oldest old ages, by contrast, the differences were more pronounced when socioeconomic position was measured in terms of education and occupational social class. Nevertheless, inequalities in dementia mortality emerged substantially later in life compared to mortality from all other causes. Socioeconomic inequalities in mortality from other causes diminished after the age of 95, whereas inequalities in dementia mortality in terms of education persisted to the oldest old age and even increased after the age of 95.

[Insert Figure 1 about here]

Table 2 shows that dementia contributed to 25–30% of educational and household income differences in overall mortality at the age of 70+. Dementia contributed less to occupational social class differences (12%). The contribution of dementia to the excess mortality in the low educational and household income groups increased substantially with age.

[Insert Table 2 about here]

All indicators of midlife socioeconomic position were associated with dementia mortality in Cox regression models (Table 3, model 1). The associations were strongest for basic education (hazard ratio [HR]=1.23, 95% CI 1.15–1.32), unknown occupational social class (HR=1.20, 1.00–1.44), and the lowest household income quintile (HR=1.28, 1.20–1.35). Mutual adjustment of socioeconomic indicators in model 2 attenuated educational differences by about 40%, and unknown occupational social class to a non-significant level. Basic education (HR=1.14, 1.06–1.23), manual occupational social class (HR=1.06, 1.01–1.11) and three lowest household income quintiles (for the lowest quintile HR=1.24, 1.16–1.32) all

For peer review only - http://bmjopen.bmj.com/site/about/guidelines.xhtml

predicted dementia mortality independently of each other. Adjustment for midlife economic activity in model 3 attenuated the excess hazard particularly of the lower household income quintiles. Adjustment for baseline marital status and chronic health conditions in model 4 contributed to a small change in the estimates, the attenuation being largest for the lowest household income quintile. In this full model, basic education increased the hazard of dementia death by 14% (1.05–1.22), manual occupational social class by 5% (1.00–1.11) and the two lowest household income quintiles by 7–13% (HR=1.07, 1.00–1.14 to HR=1.13, 1.06–1.21).

[Insert Table 3 about here]

Discussion

Main findings and their interpretation

In this study we have shown that dementia mortality at older ages is socioeconomically patterned. People with lower education, occupational social class and household income have a higher risk of dementia death compared to those with higher socioeconomic position. These results add to the literature on socioeconomic inequalities in old-age mortality, which have previously shown a socioeconomic pattern in many other specific causes of death such as cardiovascular diseases, COPD and cancer (1). Our results indicate, moreover, that dementia is an important factor in overall socioeconomic inequalities in old-age mortality, contributing to 25–30% of educational and household income differences in total mortality among the population aged 70 and over. The contribution of dementia to overall socioeconomic inequalities in mortality increased substantially with age, which relates to the fact that the proportion of deaths attributable to dementia also increases with age (19).

A major difference in the patterns between dementia morality and mortality from all other causes of death was that socioeconomic inequalities in dementia mortality emerged later, were smaller and persisted in the same magnitude to the oldest old ages. Inequalities in mortality from other causes of death, instead, tended to diminish after the age of 95. The attenuation of socioeconomic inequalities with age is a general finding

For peer review only - http://bmjopen.bmj.com/site/about/guidelines.xhtml

(1,2), and may partly relate to selective survival, suggesting that people who survive to very old age have more similar health profiles across socioeconomic groups. Our results show, however, that even among people who survive to the oldest old age, socioeconomic groups differ in neurological health. This is a novel finding in that previous studies have identified consistent socioeconomic inequalities in dementia mortality only among the younger old (7,8) but the results have been mixed for the oldest old (8,9). Participation bias may at least partly explain the differences in findings; people of older age, lower socioeconomic position and health problems are less likely to participate in surveys and studies involving health examinations. Our study employed register data on a population-based cohort and thus is not affected by participation or attrition biases.

The age patterns in dementia mortality differed between indicators of socioeconomic position: while educational and occupational social class differences were more pronounced among the oldest old, the differences among the younger old were largest when socioeconomic position was measured in terms of household income. The lowest midlife household income quintiles represent the most disadvantaged population groups with multiple dementia risk factors. A low household income may be accompanied by impoverished living conditions and greater psychosocial stress, increasing the risk of chronic diseases directly or through less favourable health behaviours. Our findings show that the higher dementia mortality of the lowest midlife household income quintiles was strongly related to greater morbidity and early retirement of these groups. We cannot rule out the possibility, however, that people who died from dementia at younger old ages experienced cognitive decline already in midlife to the extent that affected their labour market participation and household income.

Education, in turn, may have particular benefits above and beyond physical health factors among the population surviving to the oldest old age. Our results show widening educational differences in dementia mortality after the age of 95, and the association was not related to chronic health conditions or marital status at baseline. Education is a well-established predictor of dementia incidence (20), although the exact mechanisms are still not known. Brain autopsy studies indicate, in line with the cognitive reserve hypothesis

For peer review only - http://bmjopen.bmj.com/site/about/guidelines.xhtml

(21), that education is not associated with the burden of neuropathology at death but higher education enables individuals to compensate longer for the neuropathological changes before developing clinical symptoms of dementia (22). Thus, it is possible that the educational differences in dementia mortality we found in our study are due to competing risks; people with higher education died from other causes before they reached the phase of clinical dementia or died from other causes before dementia progressed to death. However, the empirical evidence for the cognitive reserve hypothesis remains open to debate. For example, multiple studies have not identified educational differences in survival time after dementia onset (23), which is among the key hypotheses in the cognitive reserve model (24). Therefore, it is plausible that higher education truly enhances brain health and protects against the development of neurodegenerative disorders.

We used a unique population-representative sample of older adults in Finland with 11,395 dementia deaths identified from the National Death Register. The register-based sample was not affected by participation or attrition bias, which are common limitations of many cohort designs, particularly among the older population. The population register encompasses rich information on demographic and socioeconomic characteristics of individuals over the life course, and is not subject to bias from individuals' self-reports or recollection.

Despite the rich register data, our study also has some limitations. First, we could only identify cases that have been recorded on the death certificate. To minimise any bias arising from potential underreporting of dementia as the underlying cause of death, we applied the multiple-cause approach and included also cases where dementia was recorded as any of the three contributory causes (25). According to a validation study for identifying dementia and Alzheimer's disease in the Finnish national registers, the documentation of dementia as the cause of death has improved since the late 1990s, and the specificity is particularly high (26). Furthermore, we ran sensitivity analyses with interaction with calendar year, and found that the associations between the indicators of socioeconomic position and dementia mortality did not vary in time. Therefore, we believe our results are not biased by possible changes in documentation practices.

For peer review only - http://bmjopen.bmj.com/site/about/guidelines.xhtml

Second, the causal relationship between socioeconomic position and dementia is difficult to establish in observational studies. We therefore measured all socioeconomic characteristics 15–30 years before the mortality follow-up, and it is thus very unlikely that any symptoms of dementia affected the midlife socioeconomic attainment of individuals. Nevertheless, we cannot exclude the possibility that early cognitive decline may have affected midlife socioeconomic position, especially measured in term of occupational social class and household income.

Conclusions

This study provides new insight into the socioeconomic inequalities in old-age mortality by showing a consistent socioeconomic pattern in dementia mortality that persists to the oldest old ages. Low education, occupational social class and household income were all associated with higher risk of dementia death, although the socioeconomic differences emerged later and were smaller than in mortality from other causes. Household income differences in dementia mortality were more pronounced among the younger old, and the associations were largely attributable to other chronic health conditions such as diabetes and stroke. Educational inequalities, by contrast, were independent of chronic health conditions and became more pronounced at the oldest old age where mortality inequalities generally begin to attenuate. The findings suggest that dementia contributes to socioeconomic inequalities in overall mortality at older ages and, thus, dementia prevention is important also from the point of view of socioeconomic inequalities in mortality.

For peer review only

For peer review only - http://bmjopen.bmj.com/site/about/guidelines.xhtml

REFERENCES

1. Huisman M, Kunst AE, Bopp M, et al. Educational inequalities in cause-specific mortality in middle-aged and older men and women in eight western European populations. *The Lancet*. 2005;365(9458):493–500.

2. Huisman M, Read S, Towriss CA, et al. Socioeconomic Inequalities in Mortality Rates in Old Age in the World Health Organization Europe Region. *Epidemiol Rev*. 2013;35(1):84–97.

3. Marmot MG, Shipley MJ. Do socioeconomic differences in mortality persist after retirement? 25 Year follow up of civil servants from the first Whitehall study. *BMJ*. 1996;313(7066):1177–1180.

4. Demakakos P, Biddulph JP, Bobak M, et al. Wealth and mortality at older ages: a prospective cohort study. *J Epidemiol Community Health*. 2016;70(4):346–353.

5. Prince M, Wimo A, Guerchet M, et al. World Alzheimer Report 2015—The Global Impact of Dementia: an analysis of prevalence, incidence, cost and trends. 2015;(https://www.alz.co.uk/research/WorldAlzheimerReport2015.pdf). (Accessed September 13, 2017)

6. Office for National Statistics. Deaths registered in England and Wales (series DR): 2016. 2017;(https://www.ons.gov.uk/peoplepopulationandcommunity/birthsdeathsandmarriages/deaths/bulletins/deathsregisteredinenglandandwalesseriesdr/2016#dementia-and-alzheimer-disease-remained-the-leading-cause-of-death-in-2016)

7. Russ TC, Stamatakis E, Hamer M, et al. Socioeconomic status as a risk factor for dementia death: individual participant meta-analysis of 86 508 men and women from the UK. *The British journal of psychiatry*. 2013;203(1):10–17.

8. Strand BH, Langballe EM, Rosness TA, et al. Age, education and dementia related deaths. The Norwegian Counties Study and the Cohort of Norway. *Journal of the neurological sciences*. 2014;345(1):75–82.

9. Enroth L, Raitanen J, Hervonen A, et al. Is socioeconomic status a predictor of mortality in nonagenarians? The vitality 90+ study. *Age and ageing*. 2014;44(1):123–129.

10. Martelin T. Mortality by indicators of socioeconomic status among the Finnish elderly. *Social science & medicine*. 1994;38(9):1257–1278.

11. Martikainen P, Blomgren J, Valkonen T. Change in the total and independent effects of education and occupational social class on mortality: analyses of all Finnish men and women in the period 1971–2000. *Journal of Epidemiology & Community Health*. 2007;61(6):499–505.

12. Rognerud MA, Zahl P-H. Social inequalities in mortality: changes in the relative importance of income, education and household size over a 27-year period. *The European Journal of Public Health*. 2005;16(1):62–68.

13. Geyer S, Hemström Ö, Peter R, et al. Education, income, and occupational class cannot be used interchangeably in social epidemiology. Empirical evidence against a common practice. *Journal of Epidemiology & Community Health*. 2006;60(9):804–810.

For peer review only

For peer review only - http://bmjopen.bmj.com/site/about/guidelines.xhtml

14. Strand BH, Skirbekk V, Rosness TA, et al. Income in midlife and dementia related mortality over three decades: A Norwegian prospective study. *eNeurologicalSci*. 2015;1(2):24–29.

15. OECD. OECD Framework for Statistics on the Distribution of Household Income, Consumption and Wealth. Paris: OECD Publishing; 2013 (Accessed May 3, 2018).(http://dx.doi.org/10.1787/9789264194830-en). (Accessed May 3, 2018)

16. Baumgart M, Snyder HM, Carrillo MC, et al. Summary of the evidence on modifiable risk factors for cognitive decline and dementia: a population-based perspective. *Alzheimer's & dementia: the journal of the Alzheimer's Association*. 2015;11(6):718–726.

17. Lamarca R, Alonso J, Gómez G, et al. Left-truncated data with age as time scale: an alternative for survival analysis in the elderly population. *The Journals of Gerontology Series A: Biological Sciences and Medical Sciences*. 1998;53(5):M337–M343.

18. StataCorp. Stata Statistical Software: Release 15. College Station, TX: StataCorp LLC. 2017;

19. Knapp M, Prince M, Albanese E, et al. Dementia UK: The full report. London: Alzheimer's Society; 2007.

20. Livingston G, Sommerlad A, Orgeta V, et al. Dementia prevention, intervention, and care. *The Lancet* [electronic article]. 2017;(https://tbhtoolkits.com/media/Dementia-Prevention-Intervention-and-Care-Lancet-2017-1.pdf). (Accessed September 11, 2017)

21. Stern Y. What is cognitive reserve? Theory and research application of the reserve concept. *Journal of the International Neuropsychological Society*. 2002;8(3):448–460.

22. Brayne C, Ince PG, Keage HA, et al. Education, the brain and dementia: neuroprotection or compensation? EClipSE Collaborative Members. *Brain*. 2010;133(8):2210–2216.

23. Paradise M, Cooper C, Livingston G. Systematic review of the effect of education on survival in Alzheimer's disease. *International psychogeriatrics*. 2009;21(1):25–32.

24. Stern Y, Tang MX, Denaro J, et al. Increased risk of mortality in Alzheimer's disease patients with more advanced educational and occupational attainment. *Annals of Neurology: Official Journal of the American Neurological Association and the Child Neurology Society*. 1995;37(5):590–595.

25. Garcia-Ptacek S, Kåreholt I, Cermakova P, et al. Causes of Death According to Death Certificates in Individuals with Dementia: A Cohort from the Swedish Dementia Registry. *Journal of the American Geriatrics Society*. 2016;64(11).

26. Solomon A, Ngandu T, Soininen H, et al. Validity of dementia and Alzheimer's disease diagnoses in Finnish national registers. *Alzheimer's & Dementia*. 2014;10(3):303–309.

For peer review only - http://bmjopen.bmj.com/site/about/guidelines.xhtml

Footnotes

Contributors: All authors participated in designing the study, generating hypotheses, interpreting the data and critically revised the manuscript for important intellectual content. KK analysed the data, conducted the literature review and wrote the first draft of the manuscript.

Funding: KK was supported by the Eino Jutikkala Fund. PM was supported by the Academy of Finland, and the Strategic Research Council PROMEQ project (#303615). PM was also supported by the European Union Horizon2020 Programme under grant agreement n° 667661 (Promoting mental wellbeing in the ageing population - MINDMAP). The study does not necessarily reflect the Commission's views and in no way anticipates the Commission's future policy in this area. The funders had no role in study design, data collection and analysis, decision to publish, or preparation of the manuscript.

Competing interests: None declared.

Data sharing statement: Statistics Finland, the National Institute for Health and Welfare and the Social Insurance Institution of Finland have collected and own the data. Due to data protection regulations, the authors are not allowed to make the data available to third parties. Researchers can apply for data access by contacting the register-holding institutions: Statistics Finland (http://stat.fi/index_en.html); National Institute for Health and Welfare (https://thl.fi/en); Social Insurance Institution of Finland (https://www.kela.fi/web/en).

Ethics approval: The study has been approved by Statistics Finland Board of Ethics (permit TK-53-339-13). The data were collected for routine administrative registration purposes and, therefore, informed consent of the participants was not obtained. These register data can be used for scientific purposes under the Personal Data Act and the Statistics Act. Statistics Finland anonymised the data prior to providing them to researchers.

For peer review only

Table 1. Distribution of the study population, dementia deaths and age-adjusted dementia mortality rates (per 10,000 person-years) by indicators of socioeconomic position and other characteristics, Finnish men and women in 2001–2016

			Dementia deaths		
	**N**	**%**	**n**	**Rate**	**95% CI**
Mean age at baseline (SD)	76.4 (4.8)				
Gender					
Men	20100	36.6	3409	191.2	184.9–197.8
Women	34864	63.4	7986	228.1	223.1–233.2
Education					
Tertiary	5445	9.9	1014	178.7	168.0–190.1
Secondary	7074	12.9	1446	200.3	190.2–210.9
Basic	42445	77.2	8936	223.7	219.1–228.4
Occupational social class					
White-collar	17015	31.0	3524	200.1	193.6–206.8
Manual	23951	43.6	4882	216.8	210.8–223.0
Farmer	10204	18.6	2211	242.0	232.1–252.3
Other self-employed	3271	6.0	657	208.6	193.2–225.1
Unknown	523	1.0	121	289.2	242.0–345.6
Household income					
Highest quintile	13667	24.9	2715	194.0	186.9–201.5
2nd	10522	19.1	2098	201.9	193.5–210.8
3rd	10110	18.4	2114	217.5	208.4–227.0
4th	10292	18.7	2183	224.3	215.1–233.9
Lowest quintile	10373	18.9	2285	253.8	243.6–264.4
Economic activity					
Active	37266	67.8	7585	208.1	203.4–212.8
Retired	8881	16.2	1742	219.2	209.1–229.7
Other inactive	8817	16.0	2068	245.2	234.9–256.0
Marital status					
Married	24789	45.1	4471	175.5	170.4–180.7
Divorced	4056	7.4	797	204.6	190.8–219.3
Widowed	20997	38.2	5000	265.8	258.5–273.2
Never married	5122	9.3	1127	242.3	228.6–256.9
Chronic health conditions					
Alcohol-related diseases	308	0.6	68	329.9	260.1–418.4
Asthma and COPD	4510	8.2	789	206.9	192.9–221.8
Diabetes	6714	12.2	1240	243.7	230.5–257.6
Heart disease	18094	32.9	3562	244.5	236.6–252.6

For peer review only - http://bmjopen.bmj.com/site/about/guidelines.xhtml

Stroke	3180	5.8	685	328.3	304.6–353.8
Region of residence					
Western Finland	25078	45.6	4979	206.0	200.3–211.8
Helsinki capital region	7449	13.6	1582	218.0	207.5–229.0
Rest of Southern Finland	12056	21.9	2464	213.8	205.6–222.5
Eastern Finland	8458	15.4	1916	238.9	228.4–249.8
Lapland	1923	3.5	454	243.6	222.2–267.1
Degree of urbanisation					
Urban	29853	51.0	6401	221.3	215.9–226.8
Semi-urban	9285	17.7	1831	205.4	196.2–215.0
Rural	15826	31.3	3163	210.9	203.7–218.4
Total	54964	100.0	11395	215.7	211.7–219.7

Abbreviations: CI, confidence intervals; COPD, chronic obstructive pulmonary diseases

Table 2. The contribution (%) of dementia to socioeconomic differences in total mortality by indicator of socioeconomic position and age, Finnish men and women in 2001–2016

	70-79	80-89	90+	All ages 70+
Education[a]	8.6	31.7	52.9	29.9
Occupational social class[b]	6.4	19.5	19.8	12.0
Household income[c]	13.3	22.2	35.0	24.0

[a] Tertiary vs. basic education

[b] White-collar vs. manual occupational social class

[c] Highest vs. lowest household income quintiles

For peer review only - http://bmjopen.bmj.com/site/about/guidelines.xhtml

Table 2. Hazard ratios and 95% confidence intervals for dementia mortality by indicators of socioeconomic position, Finnish men and women in 2001–2016, n=54,964

Indicator of socioeconomic position	Model 1[a]		Model 2[b]		Model 3[c]		Model 4[d]	
	HR	95% CI	HR	95% CI	HR	95% CI	HR	95% CI
Education								
Tertiary	1.00		1.00		1.00		1.00	
Secondary	1.14	1.05–1.23	1.08	0.99–1.17	1.08	0.99–1.17	1.08	0.99–1.17
Basic	1.23	1.15–1.32	1.14	1.06–1.23	1.14	1.05–1.22	1.14	1.05–1.22
Occupational social class								
White collar	1.00		1.00		1.00		1.00	
Manual	1.14	1.09–1.20	1.06	1.01–1.11	1.05	1.00–1.11	1.05	1.00–1.11
Farmer	1.08	1.02–1.15	0.96	0.90–1.03	0.97	0.91–1.04	0.98	0.92–1.05
Other self-employed	1.05	0.96–1.14	0.98	0.90–1.07	0.99	0.91–1.08	1.00	0.92–1.09
Unknown	1.20	1.00–1.44	1.04	0.87–1.25	0.94	0.78–1.14	0.94	0.78–1.14
Household income								
Highest quintile	1.00		1.00		1.00		1.00	
2nd	1.08	1.02–1.14	1.04	0.98–1.10	1.03	0.98–1.10	1.02	0.96–1.09
3rd	1.13	1.07–1.20	1.08	1.02–1.15	1.07	1.00–1.14	1.05	0.99–1.12
4th	1.17	1.10–1.24	1.13	1.06–1.20	1.10	1.03–1.17	1.07	1.00–1.14
Lowest quintile	1.28	1.20–1.35	1.24	1.16–1.32	1.18	1.10–1.26	1.13	1.06–1.21

Abbreviations: CI, confidence interval; HR, hazard ratio

All models used age as time scale and adjusted for calendar year, gender, region of residence and the degree of urbanisation

[a]Model 1: each indicator of socioeconomic position separately

[b]Model 2: indicators of socioeconomic position mutually adjusted

[c]Model 3: model 2 + midlife economic activity

[d]Model 4: model 3 + baseline marital status and chronic health conditions (alcohol-related diseases, asthma and chronic obstructive pulmonary disease, diabetes, heart disease and stroke)

For peer review only - http://bmjopen.bmj.com/site/about/guidelines.xhtml

Figure 1. Kaplan–Meier survival probabilities for dementia mortality and mortality from all other causes of death by a) education, b) occupational social class and c) household income quintile (Q1=highest, Q5=lowest), Finnish men and women in 2001–2012

For peer review only

For peer review only - http://bmjopen.bmj.com/site/about/guidelines.xhtml

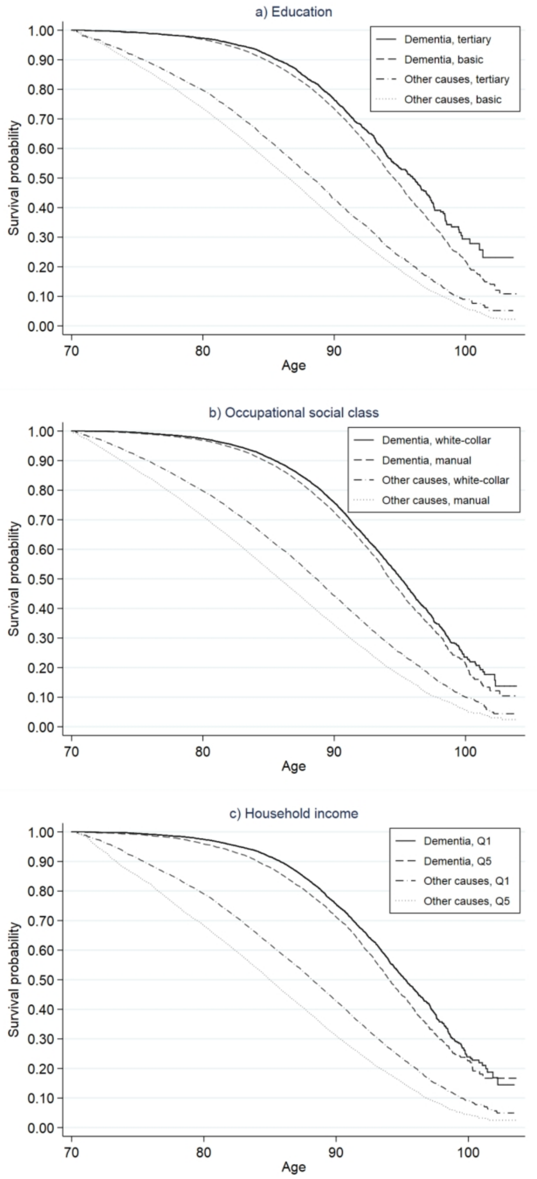

Figure 1. Kaplan–Meier survival probabilities for dementia mortality and mortality from all other causes of death by a) education, b) occupational social class and c) household income quintile (Q1=highest, Q5=lowest), Finnish men and women in 2001–2012

Supplementary Table 1 Differences in mortality rates (per 10,000 person-years) by cause of death, and the contribution of each cause of death (%) to differences in total mortality by indicator of socioeconomic position and age

	70–79		80–89		90+	
	Rate difference	Contribution (%)	Rate difference	Contribution (%)	Rate difference	Contribution (%)
Education[a]						
Dementia	9.1	8.6	40.4	31.7	152.4	52.9
Other causes	97.3	91.4	86.9	68.3	135.6	47.1
Total mortality	106.4	100.0	127.2	100.0	288.0	100.0
Occupational social class[b]						
Dementia	7.8	6.4	33.0	19.5	49.1	19.8
Other causes	114.2	93.6	136.1	80.5	199.3	80.2
Total mortality	122.0	100.0	169.1	100.0	248.5	100.0
Household income[c]						
Dementia	23.5	13.3	49.4	22.2	127.8	35.0
Other causes	152.9	86.7	173.3	77.8	237.4	65.0
Total mortality	176.4	100.0	222.7	100.0	365.2	100.0

[a] Tertiary vs. basic education

[b] White-collar vs. manual occupational social class

[c] Highest vs. lowest household income quintiles

STROBE Statement—Checklist of items that should be included in reports of ***cohort studies***

	Item No	Recommendation	Page No
**Title and abstract**	1	(*a*) Indicate the study's design with a commonly used term in the title or the abstract	1
		(*b*) Provide in the abstract an informative and balanced summary of what was done and what was found	2-3
**Introduction**			
Background/rationale	2	Explain the scientific background and rationale for the investigation being reported	4
Objectives	3	State specific objectives, including any prespecified hypotheses	5
**Methods**			
Study design	4	Present key elements of study design early in the paper	5-6
Setting	5	Describe the setting, locations, and relevant dates, including periods of recruitment, exposure, follow-up, and data collection	5-6
Participants	6	(*a*) Give the eligibility criteria, and the sources and methods of selection of participants. Describe methods of follow-up	5-6
		(*b*) For matched studies, give matching criteria and number of exposed and unexposed	
Variables	7	Clearly define all outcomes, exposures, predictors, potential confounders, and effect modifiers. Give diagnostic criteria, if applicable	6-7
Data sources/ measurement	8*	For each variable of interest, give sources of data and details of methods of assessment (measurement). Describe comparability of assessment methods if there is more than one group	6-7
Bias	9	Describe any efforts to address potential sources of bias	8-9
Study size	10	Explain how the study size was arrived at	5-6
Quantitative variables	11	Explain how quantitative variables were handled in the analyses. If applicable, describe which groupings were chosen and why	6-9
Statistical methods	12	(*a*) Describe all statistical methods, including those used to control for confounding	8-9
		(*b*) Describe any methods used to examine subgroups and interactions	9
		(*c*) Explain how missing data were addressed	5-6
		(*d*) If applicable, explain how loss to follow-up was addressed	8
		(*e*) Describe any sensitivity analyses	13
**Results**			
Participants	13*	(a) Report numbers of individuals at each stage of study—eg numbers potentially eligible, examined for eligibility, confirmed eligible, included in the study, completing follow-up, and analysed	9
		(b) Give reasons for non-participation at each stage	9
		(c) Consider use of a flow diagram	
Descriptive data	14*	(a) Give characteristics of study participants (eg demographic, clinical, social) and information on exposures and potential confounders	9
		(b) Indicate number of participants with missing data for each variable of interest	
		(c) Summarise follow-up time (eg, average and total amount)	
Outcome data	15*	Report numbers of outcome events or summary measures over time	9

For peer review only - http://bmjopen.bmj.com/site/about/guidelines.xhtml

Main results	16	(*a*) Give unadjusted estimates and, if applicable, confounder-adjusted estimates and their precision (eg, 95% confidence interval). Make clear which confounders were adjusted for and why they were included<br>(*b*) Report category boundaries when continuous variables were categorized<br>(*c*) If relevant, consider translating estimates of relative risk into absolute risk for a meaningful time period	9-11
Other analyses	17	Report other analyses done—eg analyses of subgroups and interactions, and sensitivity analyses	9
**Discussion**			
Key results	18	Summarise key results with reference to study objectives	11-12
Limitations	19	Discuss limitations of the study, taking into account sources of potential bias or imprecision. Discuss both direction and magnitude of any potential bias	13-14
Interpretation	20	Give a cautious overall interpretation of results considering objectives, limitations, multiplicity of analyses, results from similar studies, and other relevant evidence	11-13
Generalisability	21	Discuss the generalisability (external validity) of the study results	13
**Other information**			
Funding	22	Give the source of funding and the role of the funders for the present study and, if applicable, for the original study on which the present article is based	17

*Give information separately for exposed and unexposed groups.

Note: An Explanation and Elaboration article discusses each checklist item and gives methodological background and published examples of transparent reporting. The STROBE checklist is best used in conjunction with this article (freely available on the Web sites of PLoS Medicine at http://www.plosmedicine.org/, Annals of Internal Medicine at http://www.annals.org/, and Epidemiology at http://www.epidem.com/). Information on the STROBE Initiative is available at http://www.strobe-statement.org.

For peer review only

For peer review only - http://bmjopen.bmj.com/site/about/guidelines.xhtml

BMJ Open

Midlife socioeconomic position and old-age dementia mortality: a large prospective register-based study from Finland

Journal:	*BMJ Open*
Manuscript ID	bmjopen-2019-033234.R1
Article Type:	Original research
Date Submitted by the Author:	27-Sep-2019
Complete List of Authors:	Korhonen, Kaarina; University of Helsinki, Population Research Unit<br>Einiö, Elina; University of Helsinki<br>Leinonen, Taina; Finnish Institute of Occupational Health,<br>Tarkiainen , Lasse ; University of Helsinki<br>Martikainen, Pekka; University of Helsinki
**Primary Subject Heading**:	Neurology
Secondary Subject Heading:	Public health, Sociology
Keywords:	Dementia < NEUROLOGY, Socioeconomic factors, Alzheimer's disease, Register study

SCHOLARONE™
Manuscripts

Midlife socioeconomic position and old-age dementia mortality: a large prospective register-based study from Finland

Kaarina Korhonen[1], Elina Einiö[1,2,3], Taina Leinonen[4], Lasse Tarkiainen[1], Pekka Martikainen[1, 3, 5]

[1] Population Research Unit, Faculty of Social Sciences, University of Helsinki, Helsinki, Finland
[2] Department of Social Policy, London School of Economics and Political Science, London, United Kingdom
[3] Max Planck Institute for Demographic Research, Rostock, Germany
[4] Finnish Institute of Occupational Health, Helsinki, Finland
[5] Department of Public Health Sciences, Stockholm University, Stockholm, Sweden

Correspondence to:
Kaarina Korhonen
Faculty of Social Sciences
P.O. Box 18, 00014 University of Helsinki, Finland
+358 50 3199 388
kaarina.korhonen@helsinki.fi

Word count: 4033

For peer review only

For peer review only - http://bmjopen.bmj.com/site/about/guidelines.xhtml

Abstract

Objectives To assess the association between multiple indicators of socioeconomic position and dementia-related death, and to estimate the contribution of dementia to socioeconomic differences in overall mortality at older ages.

Design Prospective population-based register study.

Setting Finland.

Participants 11% random sample of the population aged 70–87 resident in Finland at the end of year 2000 (N=54 964).

Main outcome measure Incidence rates, Kaplan-Meier survival probabilities and Cox regression hazard ratios of dementia mortality in 2001–2016 by midlife education, occupational social class and household income measured at ages 53–57 years.

Results During the 528 387 person-years at risk, 11 395 individuals died from dementia (215.7 per 10 000 person-years). Lower midlife education, occupational social class and household income were associated with higher dementia mortality, and the differences persisted to the oldest old ages. Compared to mortality from all other causes, however, the socioeconomic differences emerged later. Dementia accounted for 28% of the difference between low and high education groups in overall mortality at age 70+, and for 21% of the difference between lowest and highest household income quintiles. All indicators of socioeconomic position were independently associated with dementia mortality, low household income being the strongest independent predictor (HR=1.24, 95% confidence interval 1.16–1.32), followed by basic education (HR=1.14, 1.06–1.23). Manual occupational social class was related to a 6% higher hazard (HR=1.06, 1.01–1.11) compared to non-manual social class. Adjustment for midlife economic activity, baseline marital status and chronic health conditions attenuated the excess hazard of low midlife household income, although significant effects remained.

For peer review only - http://bmjopen.bmj.com/site/about/guidelines.xhtml

Conclusion Several indicators of socioeconomic position predict dementia mortality independently and socioeconomic inequalities persist into the oldest old ages. The results demonstrate that dementia is among the most important contributors to socioeconomic inequalities in overall mortality at older ages.

Strengths and limitations of this study

- We used longitudinal registry data that permits a 15-year follow-up of dementia mortality with no attrition or recall bias.
- Dementia is documented in the national death register with high specificity.
- Due to the use of register data, traditional dementia risk factors such as smoking and physical activity could not be measured.
- All indicators of socioeconomic position were measured in midlife in order to avoid selection to socioeconomic groups on the basis of cognitive decline.
- This is the first study to show the contribution of dementia to the socioeconomic inequalities in overall mortality at older ages.

For peer review only

For peer review only - http://bmjopen.bmj.com/site/about/guidelines.xhtml

Introduction

Socioeconomic inequality in health and mortality is one of the most consistent findings in the demographic and social epidemiological literature. Lower education, occupational social class and income are strong predictors of all-cause and cause-specific mortality particularly among the working-age population, but inequalities are clear also at older ages.[1–4] Among the ageing population, the key factors affecting morbidity and disability are Alzheimer's disease and other forms of progressive dementia. Globally, an estimated 47 million people lived with dementia in 2015, and the number is projected to triple by 2050.[5] In England and Wales, dementia has already become the leading cause of death.[6] Despite the growing societal impact, however, no comprehensive understanding exists about the socioeconomic patterns of dementia mortality.

Educational inequalities in dementia mortality have previously been reported in studies following individuals from midlife or younger old ages[7,8] but not among the oldest old.[8,9] In a Norwegian health examination study, an educational pattern was present only among cohorts aged below 70 at baseline but not among those aged 70 and over.[8] Similarly, among a Finnish cohort aged 90 and over, no statistically significant educational gradient in dementia mortality emerged.[9] The lack of educational differentials among the oldest old may relate to selective survival. People with lower education experience higher mortality at younger ages, and those who survive to older ages do so because of their better health. Thus, the population surviving to older ages is more homogeneous in terms of health-related characteristics and, as a result, the socioeconomic differences in mortality are diminished. Another possible explanation for the lack of educational gradient in dementia mortality is the fact that the distribution of education in the oldest cohorts is highly skewed. Given that the majority of people in these cohorts have no more than basic education, other indicators of socioeconomic position (SEP) may be more suitable for identifying high-risk population subgroups.[2,10] Previous studies suggest that among adults in general, overall mortality disparities are greater or have increased to a greater extent in terms of occupational social class[11] and income[12,13] than education. Among the Finnish cohort of nonagenarians,[9] occupational social class

For peer review only - http://bmjopen.bmj.com/site/about/guidelines.xhtml

was a strong predictor of dementia mortality with a 3-fold hazard of dementia death among the unskilled manual workers compared to upper non-manuals. Personal income in midlife, however, was not related to dementia mortality among a cohort of Norwegian men.[14] To our knowledge, no previous study has assessed inequalities in dementia mortality by household income, a socioeconomic indicator that is more directly related to material resources available to the individual and that more rigorously captures the living conditions of the most disadvantaged population subgroups. A low household income may, in addition to material disadvantage, induce psychosocial stress, increasing the risk of dementia directly or through less favourable health behaviours. Disentangling the contributions of education, occupational social class and household income will thus provide important insights into the potential mechanisms how SEP shapes the risk of dementia death.

This study contributes to the existing knowledge by assessing socioeconomic inequalities in dementia mortality using multiple indicators of SEP, including education, occupational social class and household income. More specifically, the aims of the study were to 1) investigate the magnitude of socioeconomic inequalities in dementia mortality in relation to age, and compare the patterns to those in mortality from all other causes of death, 2) to quantify the contribution of dementia to the socioeconomic inequalities in overall mortality at older ages, and 3) to assess whether education, occupational social class and household income are independently related to dementia mortality once the other indicators are taken into account. This was because different indicators of SEP are correlated but each of them may have independent associations with dementia mortality. We further estimated models adjusting for other risk factors including marital status and various chronic health conditions. We used longitudinal registry data on a large population-based sample, which permits a 15-year follow-up of dementia-related deaths with no attrition or recall bias. All indicators of SEP were measured in midlife in order to avoid selection to socioeconomic groups on the basis of cognitive decline.

Methods

Sample

For peer review only - http://bmjopen.bmj.com/site/about/guidelines.xhtml

We used an 11% random sample of the Finnish population in 1987–2007 drawn from the Statistics Finland population register, which covers all permanent residents. Statistics Finland linked the sample with information from various administrative registers including the national Death Register and healthcare registers using unique personal identification numbers assigned to all permanent residents.

In the present study, we included men and women aged 70–87 at the end of year 2000. For these cohorts, midlife socioeconomic characteristics could be identified using information from the Population Censuses conducted in 1970, 1975, 1980 and 1985. Individuals with missing census information due to residing outside of Finland (n=920) and those with missing household income information due to not being part of the household population in the census year (n=401) were excluded. 7 individuals emigrated during the first year of follow-up and thus were excluded from the analyses. The analytic sample consisted of 54 964 individuals.

Mortality data

Dates and causes of death were obtained from the Death Register. Dementia-related deaths were identified using the International Classification of Diseases 10th revision (ICD-10) codes F00–03 and G30 as the underlying or any of the three contributory causes of death reported on the death certificate. We identified 11 395 persons who died from dementia and 30 637 persons who died from other causes during the follow-up in 2001–2016.

Indicators of socioeconomic position

The information of all indicators of SEP was derived from the quinquennial population censuses of 1970–1985. A particular census year was chosen on the basis of the study subject's age so that the indicators were measured at around the age of 55 (range 53–57) for all. Education was indicated as the highest achieved qualification, categorised as tertiary (generally 13+ years of education; International Standard Classification of Education ISCED-1997 codes 5–6), secondary (10–12 years, ISCED 3–4), and basic education/no qualifications (9 years, ISCED 0–2). Occupational social class comprised five groups,

For peer review only - http://bmjopen.bmj.com/site/about/guidelines.xhtml

classified as non-manual, manual, self-employed farmer, other self-employed, and no occupation/unknown. Information of occupational social class in the census year was lacking for 10 465 individuals due to non-employment at that time. For 9942 individuals, the information could nevertheless be obtained from previous years in which the individuals were employed. Household income indicated the taxable annual income of all household members, including all income received in money or monetary benefit subject to tax. The information was obtained from the Finnish Tax Administration and the Social Insurance Institution of Finland. We adjusted for household composition using the OECD-modified equivalence scale.[15] Income quintiles were formed based on the household income distribution in the population aged 15 and over in the census year.

Covariates

The analyses incorporated information of economic activity measured from the census year because being out of the labour market may indicate poor health and affect dementia risk independently but also lead to reduced household income. Economic activity was classified as being in the labour force, retired and other inactive. Marital status was measured at baseline (the end of 2000), classified as married, divorced, widowed and never married. Baseline chronic health conditions included indicators of vascular and lifestyle risk factors for dementia,[16] and were identified from health registers in the five-year period before the baseline, covering 1996–2000. We used the diagnostic records of the hospital discharge register and patient censuses of the National Institute for Health and Welfare, and the records of prescription medicine purchases and of entitlement to special reimbursement for the medication expenses for certain chronic diseases maintained by the Social Insurance Institution of Finland. We included indicators for alcohol-related diseases and accidental poisoning by alcohol, asthma and other chronic obstructive pulmonary disease (COPD), diabetes, heart disease and stroke (for coding see Supplementary Table 1). To account for potential regional variance in socioeconomic characteristics and mortality, we included dummies for region of residence (Western Finland, Helsinki capital region, rest of Southern Finland, Eastern Finland, and Lapland) and the degree of urbanisation of the municipality of residence, a variable based on the

For peer review only

For peer review only - http://bmjopen.bmj.com/site/about/guidelines.xhtml

proportion of population living in urban settlements and the population of the largest urban settlement in the municipality (urban, semi-urban and rural).

Statistical analyses

We followed the study population for dementia mortality from 1 January 2001 until 31 December 2016. Individuals were censored on the date of death, the end of the year preceding emigration, or at the end of 2016, whichever came first.

For descriptive statistics, we calculated age-adjusted dementia mortality rates per 10 000 person-years at risk by indicators of SEP and the covariates. In order to assess the magnitude of socioeconomic inequalities in relation to age, we estimated Kaplan–Meier survival functions by education, occupational social class and household income. In these analyses, we contrasted the survival functions of the highest and lowest education groups, non-manual and manual employees and the highest and lowest household income quintiles. The equality of survival functions was tested using log-rank tests. For the comparison between dementia mortality and the more general mortality patterns, separate Kaplan-Meier survival functions were estimated for mortality from all other causes of death. We also estimated hazard ratios and their 95% confidence intervals for low versus high socioeconomic groups at the age of 70–79, 80–89 and 90 years and over.

To quantify the contribution of dementia to socioeconomic differences in overall mortality at older ages, we calculated absolute rate differences in mortality between socioeconomic groups (basic vs. tertiary education, manual vs. non-manual occupational social class, lowest vs. highest household income quintile) by cause of death. The contribution was determined by the rate difference in dementia mortality as a percentage of the rate difference in total mortality. Because the level of dementia mortality increases substantially with age, we also assessed age-specific contributions (at the age of 70–79, 80–89 and 90+).

To estimate the independent associations between each indicator of SEP and dementia mortality, we used Cox regression models. Attained age in years was used as the time scale, and thus all analyses adjusted for

For peer review only - http://bmjopen.bmj.com/site/about/guidelines.xhtml

For peer review only

the confounding effect of age.[17] We first estimated crude associations between each indicator and dementia mortality, adjusting for calendar year dummies, gender, region of residence and the degree of urbanisation (model 1). Model 2 included education, occupational social class and household income as covariates, thus showing mutually adjusted associations. Midlife economic activity was adjusted for in model 3. We further adjusted for baseline marital status and chronic health conditions in model 4 to assess the extent to which these factors attenuated the relative hazard attached to each socioeconomic indicator.

We tested for interactions between gender and each socioeconomic indicator using likelihood ratio test. Interactions were statistically nonsignificant ($p>0.05$), and thus we conducted all analyses for men and women combined. We also tested for interactions of all pairwise combinations of the socioeconomic indicators, adjusting for the covariates of model 1. These interactions were all statistically nonsignificant ($p>0.05$). All analyses were performed using Stata 15.1.[18]

Patient and public involvement

No patients were involved in setting the research question or the outcome measures, nor were they involved in developing plans for design or implementation of the study. No patients were asked to advise on interpretation or writing up of results. There are no plans to disseminate the results of the research to study participants or the relevant patient community.

Results

Table 1 shows the distribution of the study population by indicators of midlife SEP, economic activity and baseline characteristics. The vast majority of individuals (77.2%) had no higher than basic education, and manual employees formed the largest occupational social class (43.6%). Higher household income quintiles were over-represented among the study population due to the higher incomes of the middle aged compared to the rest of the population and also partly because of greater mortality of the lower income groups between the time of measurement of midlife income and the baseline. During the 528 387 person-years at risk 11 395 individuals died from dementia, the average age-adjusted dementia mortality rate being 223.1 and

For peer review only - http://bmjopen.bmj.com/site/about/guidelines.xhtml

210.8 per 10 000 person-years among men and women, respectively. The rate was higher for those with lower education, occupational social class and household income, and also for the non-married and people with chronic health conditions apart from asthma and other COPD.

Kaplan–Meier survival functions in Figure 1 show that dementia mortality differed by all indicators of SEP (log rank test, $p<0.001$ for each indicator), and that the age patterns differed between the indicators (for 95% confidence intervals see Supplementary Table 2). The inequalities emerged at an earlier age when SEP was measured in terms of household income (Panel c) compared to education (Panel a) and occupational social class (Panel b). At the age of 90 years and above, by contrast, the differences were more pronounced when SEP was measured in terms of education. Nevertheless, inequalities in dementia mortality emerged substantially later in life compared to mortality from all other causes. Hazard ratios in Table 2 show that relative inequalities in mortality tended to diminish with age for all indicators of SEP regardless of cause of death. However, education differences in dementia mortality showed a different age pattern in that the point estimates indicated stable inequality with age.

Overall, dementia contributed to 28.1% of educational and 20.9% of household income differences in total mortality at the age of 70 and over (Table 2). The contribution to occupational social class differences was somewhat smaller (16.7%). The contribution of dementia to socioeconomic inequalities substantially increased from the age of 70–79 to 90 years and over.

Cox regression models in Table 3 show adjusted hazard ratios (HR) for dementia mortality across all ages from 70 years and over. Adjusted for calendar year, gender, region of residence and the degree of urbanisation in model 1, the associations were strongest for basic education (HR=1.23, 95% CI 1.15–1.32), unknown occupational social class (HR=1.20, 1.00–1.44), and the lowest household income quintile (HR=1.28, 1.20–1.35). Mutual adjustment of socioeconomic indicators in model 2 attenuated educational differences by about 40%, and unknown occupational social class to a non-significant level. Basic education (HR=1.14, 1.06–1.23), manual occupational social class (HR=1.06, 1.01–1.11) and three lowest household

For peer review only - http://bmjopen.bmj.com/site/about/guidelines.xhtml

income quintiles (for the lowest quintile HR=1.24, 1.16–1.32) all predicted dementia mortality independently of each other. Adjustment for midlife economic activity in model 3 attenuated the excess hazard particularly of the lower household income quintiles. Adjustment for baseline marital status and chronic health conditions in model 4 contributed to a small change in the estimates, the attenuation being largest for the lowest household income quintile. In this full model, basic education increased the hazard of dementia death by 14% (1.05–1.22), manual occupational social class by 5% (1.00–1.11) and the two lowest household income quintiles by 7–13% (HR=1.07, 1.00–1.14 to HR=1.13, 1.06–1.21).

Discussion

Main findings and their interpretation

In this study we have shown that dementia mortality at older ages is socioeconomically patterned in terms of multiple indicators of SEP. People with lower education, occupational social class and household income have a higher risk of dementia death compared to those with higher SEP. These results add to the literature on socioeconomic inequalities in old-age mortality, which has previously shown a socioeconomic pattern in many other specific causes of death such as cardiovascular diseases, COPD and cancer.[1] Our results indicate, moreover, that dementia is an important factor in overall socioeconomic inequalities in old-age mortality, contributing to 21–28% of household income and educational differences in total mortality among the population aged 70 and over. The contribution of dementia to overall socioeconomic inequalities in mortality increased substantially with age, which relates to the increasing proportion of deaths attributable to dementia with advancing age.[19]

A major difference in the patterns between dementia morality and mortality from all other causes of death was that socioeconomic inequalities in dementia mortality emerged later and the inequalities in dementia mortality between high and low education groups persisted in the same magnitude to the oldest old ages (90 years and above). By contrast, inequalities in mortality from other causes of death tended to diminish with age. The attenuation of socioeconomic inequalities with age is a general finding,[1,2] and may partly

For peer review only - http://bmjopen.bmj.com/site/about/guidelines.xhtml

relate to selective survival, suggesting that people who survive to very old age have more similar health profiles across socioeconomic groups. Our results show, however, that even among people who survive to the oldest old age, education groups differ in neurological health. This is a novel finding in that previous studies have identified consistent socioeconomic inequalities in dementia mortality only among the younger old[7,8] but the results have been mixed for the oldest old.[8,9] Participation bias may at least partly explain the differences in findings; people of older age, lower SEP and with health problems are less likely to participate in surveys and studies involving health examinations. Our study employed register data on a population-based cohort and thus is not affected by participation or attrition biases.

The age patterns in dementia mortality differed between indicators of SEP: while educational differences were more pronounced among the oldest old (90 years and over), the differences among the younger old (70–79 years) were largest when SEP was measured in terms of household income. The lowest midlife household income quintiles represent the most disadvantaged population groups with potentially multiple dementia risk factors. Impoverished material conditions may affect dementia risk through, for example, psychological stress[20] and health-related behaviours and cardiovascular risk factors.[16] Our findings show that the higher dementia mortality of the lowest household income quintiles was strongly – although not fully – related to greater morbidity and early retirement of these groups. It is also possible that severe health problems that were present already in midlife affected labour market participation and household incomes and thus confounded the association between income and the risk of dementia death. Future studies are needed to establish the causal relationship between these factors using formal mediation analysis techniques.

Education, in turn, may have particular benefits above and beyond physical health factors among the population surviving to the oldest old age. Our results show persistent educational differences in dementia mortality, and the association was not related to chronic health conditions or marital status at baseline. Education is a well-established predictor of dementia incidence,[21] although the exact mechanisms are still not known. Brain autopsy studies indicate, in line with the cognitive reserve hypothesis,[22] that education

For peer review only - http://bmjopen.bmj.com/site/about/guidelines.xhtml

is not associated with the burden of neuropathology at death but higher education enables individuals to compensate longer for the neuropathological changes before developing clinical symptoms of dementia.[23] Thus, it is possible that the educational differences in dementia mortality we found in our study are due to competing risks; people with higher education died from other causes before they reached the phase of clinical dementia or died from other causes before dementia progressed to death. However, the empirical evidence for the cognitive reserve hypothesis remains open to debate. For example, several studies have not identified educational differences in survival time after dementia onset,[24] which is among the key hypotheses in the cognitive reserve model.[25] Therefore, it is plausible that higher education enhances brain health and protects against (or postpones) not only the clinical symptoms but also the development of neurodegenerative disorders.

Occupational social class differences in dementia mortality were modest following adjustment for education and household income. In particular, the high hazard among those with no occupation disappeared after these adjustments indicating that this group experienced multiple socioeconomic disadvantages. The results suggest, nevertheless, that higher social class occupations may involve greater cognitive demands and intellectual engagement, and thus enhance cognitive health.[26,27] In contrast, lower class occupations or long periods of economic inactivity due to unemployment or early retirement may reduce opportunities for cognitive investment. Overall, the results of this study suggest that all three indicators of SEP are important factors in bringing about socioeconomic differences in dementia mortality, also influencing inequalities in overall mortality among the older population.

We used a unique population-representative sample of older adults in Finland with 11 395 dementia deaths identified from the National Death Register. The register-based sample was not affected by participation or attrition bias, which are common limitations of many cohort designs, particularly among the older population. The population register encompasses rich information on demographic and socioeconomic characteristics of individuals over the life course, and is not subject to bias from individuals' self-reports or recollection.

For peer review only - http://bmjopen.bmj.com/site/about/guidelines.xhtml

Despite the rich register data, our study also has some limitations. First, we could only identify dementia cases that have been recorded on the death certificate. According to a validation study for identifying dementia in the Finnish national registers, the documentation of dementia as the cause of death has improved since the late 1990s, and the specificity is particularly high.[28] To minimise any bias arising from potential underreporting of dementia as the underlying cause of death, we applied the multiple-cause approach and included also cases where dementia was recorded as any of the three contributory causes.[29] Defined this way, we identified 21% of all deaths at the age of 70 and over to be attributable to dementia. This relatively high proportion is in line with that reported in England and Wales, where dementia accounted for 19% of all deaths at the age of 80 and over.[30] Furthermore, we ran sensitivity analyses with interaction with calendar year, and found that the associations between the indicators of SEP and dementia mortality did not vary in time. Therefore, we believe our results are not biased by overreporting or underreporting of dementia as the cause of death or by changes in documentation practices. Second, register data does not cover information of traditional risk factors related to health behaviours such as smoking and physical activity. However, we included indicators of chronic conditions to measure vascular and life style risk factors for dementia.

Third, the information of household income was based on taxable income and the variable thus excludes certain monetary transfers such as housing allowance and social assistance. These means-tested sources of income may be especially relevant for people with health problems and those outside the labour market. This might lead to overestimation of the income effect. Information of disposable income was not available for years 1970–1985, but we carried out a robustness check for the correlation between taxable and disposable household incomes (as continuous variables) using the population aged 15 and over in 1995 and found the correlation to be as high as 0.97. Therefore, it is unlikely that the use of disposable income would change the ranking of individuals in the household income distribution to the extent that it would affect our main findings.

For peer review only - http://bmjopen.bmj.com/site/about/guidelines.xhtml

Finally, the causal relationship between SEP and dementia is difficult to establish in observational studies. We therefore measured all socioeconomic characteristics 15–30 years before the mortality follow-up, and it is thus very unlikely that any symptoms of dementia affected the midlife socioeconomic attainment of individuals. Nevertheless, we cannot exclude the possibility that early cognitive decline may have affected midlife SEP, especially measured in term of occupational social class and household income. Also, given the small proportion of people with tertiary education in these cohorts (10%), it is possible that this forms a select group with multiple advantages including higher childhood SEP and early cognitive ability.

Conclusions

This study provides new insight into the socioeconomic inequalities in old-age mortality by showing a consistent pattern in dementia mortality by multiple indicators of SEP. Low education, occupational social class and household income were all associated with higher risk of dementia death, although the socioeconomic differences emerged later than in mortality from other causes. Household income differences in dementia mortality were more pronounced among the younger old, and the associations were largely attributable to other chronic health conditions such as diabetes and stroke. Educational inequalities, by contrast, were independent of chronic health conditions and became more pronounced at the oldest old age where mortality inequalities generally begin to attenuate. The results indicate that dementia mortality may be amenable to socioeconomic interventions in midlife. The findings also suggest that dementia contributes to socioeconomic inequalities in overall mortality at older ages and, thus, dementia prevention is important from the point of view of socioeconomic inequalities in total mortality.

For peer review only

REFERENCES

1 Huisman M, Kunst AE, Bopp M, *et al.* Educational inequalities in cause-specific mortality in middle-aged and older men and women in eight western European populations. *The Lancet* 2005;**365**:493–500.

2 Huisman M, Read S, Towriss CA, *et al.* Socioeconomic Inequalities in Mortality Rates in Old Age in the World Health Organization Europe Region. *Epidemiol Rev* 2013;**35**:84–97.

3 Marmot MG, Shipley MJ. Do socioeconomic differences in mortality persist after retirement? 25 Year follow up of civil servants from the first Whitehall study. *BMJ* 1996;**313**:1177–80.

4 Demakakos P, Biddulph JP, Bobak M, *et al.* Wealth and mortality at older ages: a prospective cohort study. *J Epidemiol Community Health* 2016;**70**:346–353.

5 Prince M, Wimo A, Guerchet M, *et al.* World Alzheimer Report 2015—The Global Impact of Dementia: an analysis of prevalence, incidence, cost and trends. 2015. Available: https://www.alz.co.uk/research/WorldAlzheimerReport2015.pdf (accessed 13 Sep 2017).

6 Office for National Statistics. Deaths registered in England and Wales (series DR): 2016. 2017. Available:https://www.ons.gov.uk/peoplepopulationandcommunity/birthsdeathsandmarriages/deaths/bulletins/deathsregisteredinenglandandwalesseriesdr/2016#dementia-and-alzheimer-disease-remained-the-leading-cause-of-death-in-2016 (accessed 20 Sep 2019).

7 Russ TC, Stamatakis E, Hamer M, *et al.* Socioeconomic status as a risk factor for dementia death: individual participant meta-analysis of 86 508 men and women from the UK. *The British journal of psychiatry* 2013;**203**:10–17.

8 Strand BH, Langballe EM, Rosness TA, *et al.* Age, education and dementia related deaths. The Norwegian Counties Study and the Cohort of Norway. *Journal of the neurological sciences* 2014;**345**:75–82.

9 Enroth L, Raitanen J, Hervonen A, *et al.* Is socioeconomic status a predictor of mortality in nonagenarians? The vitality 90+ study. *Age and ageing* 2014;**44**:123–129.

10 Martelin T. Mortality by indicators of socioeconomic status among the Finnish elderly. *Social science & medicine* 1994;**38**:1257–1278.

11 Martikainen P, Blomgren J, Valkonen T. Change in the total and independent effects of education and occupational social class on mortality: analyses of all Finnish men and women in the period 1971–2000. *Journal of Epidemiology & Community Health* 2007;**61**:499–505.

12 Rognerud MA, Zahl P-H. Social inequalities in mortality: changes in the relative importance of income, education and household size over a 27-year period. *The European Journal of Public Health* 2005;**16**:62–68.

13 Geyer S, Hemström Ö, Peter R, *et al.* Education, income, and occupational class cannot be used interchangeably in social epidemiology. Empirical evidence against a common practice. *Journal of Epidemiology & Community Health* 2006;**60**:804–810.

14 Strand BH, Skirbekk V, Rosness TA, *et al.* Income in midlife and dementia related mortality over three decades: A Norwegian prospective study. *eNeurologicalSci* 2015;**1**:24–29.

For peer review only - http://bmjopen.bmj.com/site/about/guidelines.xhtml

15 OECD. *OECD Framework for Statistics on the Distribution of Household Income, Consumption and Wealth*. Paris: OECD Publishing 2013. Available: http://dx.doi.org/10.1787/9789264194830-en (accessed 3 May 2018).

16 Baumgart M, Snyder HM, Carrillo MC, *et al.* Summary of the evidence on modifiable risk factors for cognitive decline and dementia: a population-based perspective. *Alzheimer's & dementia: the journal of the Alzheimer's Association* 2015;**11**:718–726.

17 Lamarca R, Alonso J, Gómez G, *et al.* Left-truncated data with age as time scale: an alternative for survival analysis in the elderly population. *The Journals of Gerontology Series A: Biological Sciences and Medical Sciences* 1998;**53**:M337–M343.

18 StataCorp. Stata Statistical Software: Release 15. College Station, TX: StataCorp LLC. 2017.

19 Knapp M, Prince M, Albanese E, *et al. Dementia UK: The full report*. London: Alzheimer's Society 2007.

20 Johansson L, Guo X, Waern M, *et al.* Midlife psychological stress and risk of dementia: a 35-year longitudinal population study. *Brain* 2010;**133**:2217–2224.

21 Livingston G, Sommerlad A, Orgeta V, *et al.* Dementia prevention, intervention, and care. *The Lancet* Published Online First: 2017. Available: https://tbhtoolkits.com/media/Dementia-Prevention-Intervention-and-Care-Lancet-2017-1.pdf (accessed 11 Sep 2017).

22 Stern Y. What is cognitive reserve? Theory and research application of the reserve concept. *Journal of the International Neuropsychological Society* 2002;**8**:448–460.

23 Brayne C, Ince PG, Keage HA, *et al.* Education, the brain and dementia: neuroprotection or compensation? EClipSE Collaborative Members. *Brain* 2010;**133**:2210–2216.

24 Paradise M, Cooper C, Livingston G. Systematic review of the effect of education on survival in Alzheimer's disease. *International psychogeriatrics* 2009;**21**:25–32.

25 Stern Y, Tang MX, Denaro J, *et al.* Increased risk of mortality in Alzheimer's disease patients with more advanced educational and occupational attainment. *Annals of Neurology: Official Journal of the American Neurological Association and the Child Neurology Society* 1995;**37**:590–595.

26 Kröger E, Andel R, Lindsay J, *et al.* Is complexity of work associated with risk of dementia? The Canadian Study of Health and Aging. *American journal of epidemiology* 2008;**167**:820–830.

27 Karp A, Andel R, Parker MG, *et al.* Mentally stimulating activities at work during midlife and dementia risk after age 75: follow-up study from the Kungsholmen Project. *The American Journal of Geriatric Psychiatry* 2009;**17**:227–236.

28 Solomon A, Ngandu T, Soininen H, *et al.* Validity of dementia and Alzheimer's disease diagnoses in Finnish national registers. *Alzheimer's & Dementia* 2014;**10**:303–309. doi:10.1016/j.jalz.2013.03.004

29 Garcia-Ptacek S, Kåreholt I, Cermakova P, *et al.* Causes of Death According to Death Certificates in Individuals with Dementia: A Cohort from the Swedish Dementia Registry. *Journal of the American Geriatrics Society* 2016;**64**. doi:10.1111/jgs.14421

For peer review only - http://bmjopen.bmj.com/site/about/guidelines.xhtml

30 Office for National Statistcs. Deaths registered in England and Wales, 2016 (dataset). 2017. Available:https://www.ons.gov.uk/file?uri=/peoplepopulationandcommunity/birthsdeathsandmarriages/deaths/datasets/deathsregisteredinenglandandwalesseriesdrreferencetables/2016/drtables16.xls (accessed 20 Sep 2019).

For peer review only

For peer review only - http://bmjopen.bmj.com/site/about/guidelines.xhtml

Footnotes

Contributors: KK, EE, TL, LT and PM participated in designing the study, generating hypotheses, interpreting the data and critically revised the manuscript for important intellectual content. KK analysed the data, conducted the literature review and wrote the first draft of the manuscript.

Funding: KK was supported by the Eino Jutikkala Fund. PM was supported by the Academy of Finland, and the Strategic Research Council PROMEQ project (#303615). PM was also supported by the European Union Horizon2020 Programme under grant agreement n° 667661 (Promoting mental wellbeing in the ageing population - MINDMAP). The study does not necessarily reflect the Commission's views and in no way anticipates the Commission's future policy in this area. The funders had no role in study design, data collection and analysis, decision to publish, or preparation of the manuscript.

Competing interests: None declared.

Data sharing statement: Statistics Finland, the National Institute for Health and Welfare and the Social Insurance Institution of Finland have collected and own the data. Due to data protection regulations, the authors are not allowed to make the data available to third parties. Researchers can apply for data access by contacting the register-holding institutions: Statistics Finland (http://stat.fi/index_en.html); National Institute for Health and Welfare (https://thl.fi/en); Social Insurance Institution of Finland (https://www.kela.fi/web/en).

Ethics approval: The study has been approved by Statistics Finland Board of Ethics (permit TK-53-339-13). The data were collected for routine administrative registration purposes and, therefore, informed consent of the participants was not obtained. These register data can be used for scientific purposes under the Personal Data Act and the Statistics Act. Statistics Finland anonymised the data prior to providing them to researchers.

For peer review only

Table 1. Distribution of the study population, dementia deaths and age-adjusted dementia mortality rates (per 10 000 person-years) by indicators of midlife socioeconomic position and economic activity and baseline characteristics, Finnish men and women in 2001–2016

			Dementia deaths		
	**N**	**%**	**n**	**Rate**	**95% CI**
Mean age at baseline (SD)	76.4 (4.8)				
Gender					
Men	20100	36.6	3409	223.1	215.6–230.5
Women	34864	63.4	7986	210.8	206.3–215.4
Education[a]					
Tertiary	5445	9.9	1014	185.5	174.3–196.7
Secondary	7074	12.9	1446	205.7	195.4–216.1
Basic	42445	77.2	8936	221.8	217.3–226.3
Occupational social class[a]					
Non-manual	17015	31.0	3524	201.1	194.6–207.6
Manual	23951	43.6	4882	228.4	222.1–234.6
Self-employed farmer	10204	18.6	2211	215.7	206.9–224.6
Other self-employed	3271	6.0	657	212.2	196.3–228.1
No occupation/unknown	523	1.0	121	239.2	196.5–282.0
Household income[a]					
Highest quintile	13667	24.9	2715	196.9	189.7–204.2
2nd	10522	19.1	2098	209.6	200.9–218.4
3rd	10110	18.4	2114	217.3	208.2–226.3
4th	10292	18.7	2183	223.2	214.0–232.3
Lowest quintile	10373	18.9	2285	241.5	231.8–251.2
Economic activity[a]					
Active	37266	67.8	7585	208.1	203.6–212.7
Retired	8881	16.2	1742	257.1	245.2–269.0
Other inactive	8817	16.0	2068	212.7	203.7–221.8
Marital status					
Married	24789	45.1	4471	208.7	202.6–214.8
Divorced	4056	7.4	797	237.0	220.9–253.2
Widowed	20997	38.2	5000	214.3	208.4–220.3
Never married	5122	9.3	1127	240.9	227.2–254.7
Chronic health conditions					
Alcohol-related diseases	308	0.6	68	505.3	381.2–629.3
Asthma and COPD	4510	8.2	789	232.9	216.9–248.9
Diabetes	6714	12.2	1240	275.0	259.8–290.2

For peer review only - http://bmjopen.bmj.com/site/about/guidelines.xhtml

Heart disease	18094	32.9	3562	237.1	229.5–244.7
Stroke	3180	5.8	685	330.5	306.4–354.6
Region of residence					
Western Finland	25078	45.6	4979	204.1	198.6–209.7
Helsinki capital region	7449	13.6	1582	208.3	198.3–218.4
Rest of Southern Finland	12056	21.9	2464	214.8	206.5–223.0
Eastern Finland	8458	15.4	1916	250.1	239.2–261.0
Lapland	1923	3.5	454	261.5	238.1–285.0
Degree of urbanisation					
Urban	29853	51.0	6401	217.2	212.0–222.4
Semi-urban	9285	17.7	1831	210.6	201.2–220.0
Rural	15826	31.3	3163	215.3	208.0–222.7
Total	54964	100.0	11395	215.7	211.7–219.7

Abbreviations: CI, confidence intervals; COPD, chronic obstructive pulmonary diseases

[a] Information from the population censuses of 1970–1985, the study population being aged 53–57 years

For peer review only

For peer review only - http://bmjopen.bmj.com/site/about/guidelines.xhtml

Table 2. Relative and absolute differences in mortality between high and low socioeconomic groups[a] by cause of death and age, and contribution (%) of dementia and other causes of death to socioeconomic differences in total mortality by age, Finnish men and women in 2001–2016

	70–79 years				80–89 years				90+ years				All ages 70+
	HR	95% CI	Rate difference	Contribution (%)	HR	95% CI	Rate difference	Contribution (%)	HR	95% CI	Rate difference	Contribution (%)	Contribution (%)
Education[b]													
Dementia	1.24	0.97–1.58	8.7	8.3	1.19	1.09–1.29	40.4	33.5	1.24	1.10–1.40	157.9	51.8	28.1
Other causes	1.38	1.26–1.50	96.1	91.7	1.11	1.06–1.17	83.0	68.9	1.13	1.03–1.24	146.9	48.2	71.8
Total mortality	1.36	1.25–1.48	104.8	100.0	1.13	1.09–1.18	120.4	102.4	1.17	1.09–1.26	304.8	100.0	100.0
Occupational social class[c]													
Dementia	1.22	1.03–1.44	7.7	6.3	1.17	1.10–1.23	35.3	20.3	1.09	1.00–1.17	63.5	22.8	16.7
Other causes	1.44	1.36–1.53	114.2	93.8	1.24	1.20–1.29	138.7	79.7	1.19	1.12–1.27	215.0	77.2	83.3
Total mortality	1.41	1.34–1.49	121.8	100.1	1.22	1.19–1.26	173.9	100.0	1.15	1.09–1.21	278.4	100.0	100.0
Household income[d]													
Dementia	1.63	1.32–2.01	22.4	12.9	1.22	1.14–1.31	46.3	21.4	1.19	1.08–1.32	135.6	35.2	20.9
Other causes	1.54	1.43–1.66	151.0	87.1	1.24	1.19–1.30	169.7	78.6	1.19	1.10–1.28	249.1	64.8	79.2
Total mortality	1.55	1.44–1.67	173.3	100.0	1.24	1.19–1.29	216.0	100.0	1.19	1.12–1.26	384.7	100.0	100.0

Abbreviations: CI, confidence interval; HR, hazard ratio

Hazard ratios adjusted for calendar year. Age-adjusted incidence rates calculated as dementia deaths per 10,000 person-years at risk. Contribution of dementia determined by the rate difference in dementia mortality as a percentage of the rate difference in total mortality

[a] Information from the population censuses of 1970–1985, the study population being aged 53–57 years

[b] Tertiary vs. basic education

[c] Non-manual vs. manual occupational social class

[d] Highest vs. lowest household income quintiles

For peer review only - http://bmjopen.bmj.com/site/about/guidelines.xhtml

Table 3. Hazard ratios and 95% confidence intervals for dementia mortality by indicators of midlife socioeconomic position[a], Finnish men and women in 2001–2016, n=54,964

Indicator of socioeconomic position	Model 1[b]		Model 2[c]		Model 3[d]		Model 4[e]	
	HR	95% CI	HR	95% CI	HR	95% CI	HR	95% CI
Education								
Tertiary	1.00		1.00		1.00		1.00	
Secondary	1.14	1.05–1.23	1.08	0.99–1.17	1.08	0.99–1.17	1.08	0.99–1.17
Basic	1.23	1.15–1.32	1.14	1.06–1.23	1.14	1.05–1.22	1.14	1.05–1.22
Occupational social class								
Non-manual	1.00		1.00		1.00		1.00	
Manual	1.14	1.09–1.20	1.06	1.01–1.11	1.05	1.00–1.11	1.05	1.00–1.11
Farmer	1.08	1.02–1.15	0.96	0.90–1.03	0.97	0.91–1.04	0.98	0.92–1.05
Other self-employed	1.05	0.96–1.14	0.98	0.90–1.07	0.99	0.91–1.08	1.00	0.92–1.09
No occupation/unknown	1.20	1.00–1.44	1.04	0.87–1.25	0.94	0.78–1.14	0.94	0.78–1.14
Household income								
Highest quintile	1.00		1.00		1.00		1.00	
2nd	1.08	1.02–1.14	1.04	0.98–1.10	1.03	0.98–1.10	1.02	0.96–1.09
3rd	1.13	1.07–1.20	1.08	1.02–1.15	1.07	1.00–1.14	1.05	0.99–1.12
4th	1.17	1.10–1.24	1.13	1.06–1.20	1.10	1.03–1.17	1.07	1.00–1.14
Lowest quintile	1.28	1.20–1.35	1.24	1.16–1.32	1.18	1.10–1.26	1.13	1.06–1.21

Abbreviations: CI, confidence interval; HR, hazard ratio

All models used age as time scale and adjusted for calendar year, gender, region of residence and the degree of urbanisation

[a] Information from the population censuses of 1970–1985, the study population being aged 53–57 years

[b] Model 1: each indicator of socioeconomic position separately

[c] Model 2: indicators of socioeconomic position mutually adjusted

[d] Model 3: model 2 + midlife economic activity

[e] Model 4: model 3 + baseline marital status and chronic health conditions (alcohol-related diseases, asthma and chronic obstructive pulmonary disease, diabetes, heart disease and stroke)

For peer review only - http://bmjopen.bmj.com/site/about/guidelines.xhtml

Figure 1. Kaplan–Meier survival probabilities for dementia mortality and mortality from all other causes of death by a) education, b) occupational social class and c) household income quintile (Q1=highest, Q5=lowest), Finnish men and women in 2001–2016. Information of midlife socioeconomic position obtained from the population censuses of 1970–1985, the study population being aged 53–57 years

For peer review only

For peer review only - http://bmjopen.bmj.com/site/about/guidelines.xhtml

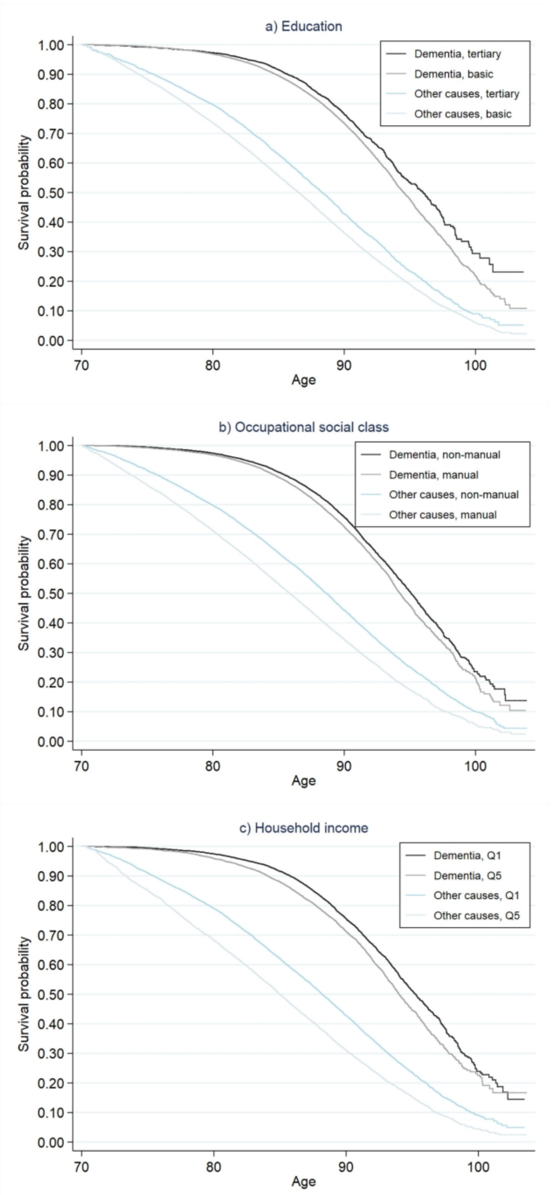

Figure 1. Kaplan–Meier survival probabilities for dementia mortality and mortality from all other causes of death by a) education, b) occupational social class and c) household income quintile (Q1=highest, Q5=lowest), Finnish men and women in 2001–2016. Information of midlife socioeconomic position obtained from the population censuses of 1970–1985, the study population being aged 53–57 years

122x265mm (300 x 300 DPI)

Supplementary Table 1. Classification of chronic health conditions used as covariates in the study

Condition	Hospital diagnoses (ICD-10)	Prescription medication (ATC)	Special reimbursement category (Finnish disease code)
Alcohol-related diseases and accidental poisoning by alcohol	F10–19, G31.2, G40.51, G62.1, G72.1, I42.6, K29.2, K70, K86.0, O35.4, X45		
Asthma and other COPD	J43–46		203
Diabetes	E10–14	A10	103
Heart disease	I00–09, I20–52		201, 206, 207
Stroke	I60–66, G45		

Abbreviations: ATC, Anatomical Therapeutic Chemical; COPD, chronic obstructive pulmonary diseases; ICD, International Classification of Diseases

view only

Supplementary Table 2. Kaplan-Meier survival probabilities at specific ages by midlife a) education, b) occupational social class and c) household income quintile (Q1=highest, Q5=lowest), Finnish men and women in 2001–2016

		Dementia		Other causes	
	Age	Survivor function	95% CI	Survivor function	95% CI
a) Education					
Tertiary	70	1.00		1.00	
	75	0.99	0.99–1.00	0.91	0.89–0.92
	80	0.97	0.97–0.98	0.80	0.78–0.82
	85	0.92	0.90–0.92	0.63	0.61–0.65
	90	0.76	0.75–0.78	0.43	0.41–0.44
	95	0.53	0.50–0.56	0.23	0.22–0.25
	100	0.29	0.24–0.35	0.09	0.07–0.11
Basic	70	1.00		1.00	
	75	0.99	0.99–1.00	0.88	0.87–0.89
	80	0.97	0.97–0.97	0.74	0.73–0.74
	85	0.89	0.89–0.90	0.56	0.55–0.56
	90	0.73	0.73–0.74	0.36	0.36–0.37
	95	0.48	0.47–0.49	0.19	0.18–0.20
	100	0.22	0.20–0.24	0.06	0.05–0.07
b) Occupational social class					
Non-manual	70	1.00		1.00	
	75	1.00	0.99–1.00	0.92	0.90–0.93
	80	0.97	0.97–0.98	0.80	0.79–0.81
	85	0.91	0.90–0.92	0.64	0.63–0.65
	90	0.76	0.75–0.77	0.44	0.43–0.45
	95	0.51	0.49–0.52	0.25	0.24–0.26
	100	0.24	0.21–0.27	0.10	0.09–0.11
Manual	70	1.00		1.00	
	75	0.99	0.99–0.99	0.87	0.85–0.88
	80	0.97	0.96–0.97	0.71	0.70–0.72
	85	0.89	0.89–0.90	0.53	0.52–0.54
	90	0.73	0.72–0.73	0.34	0.34–0.35
	95	0.46	0.45–0.47	0.17	0.17–0.18
	100	0.21	0.18–0.24	0.06	0.05–0.07

For peer review only - http://bmjopen.bmj.com/site/about/guidelines.xhtml

	Age	Dementia		Other causes	
		Survivor function	95% CI	Survivor function	95% CI
c) Household income					
Q1	70	1.00		1.00	
	75	0.99	0.99–1.00	0.91	0.90–0.92
	80	0.97	0.97–0.98	0.79	0.78–0.80
	85	0.92	0.91–0.92	0.62	0.61–0.63
	90	0.75	0.74–0.76	0.43	0.42–0.44
	95	0.51	0.49–0.53	0.24	0.22–0.25
	100	0.24	0.20–0.28	0.09	0.08–0.11
Q5	70	1.00		1.00	
	75	0.99	0.99–1.00	0.85	0.83–0.87
	80	0.96	0.95–0.97	0.68	0.67–0.70
	85	0.88	0.87–0.89	0.50	0.48–0.51
	90	0.71	0.70–0.72	0.31	0.30–0.32
	95	0.45	0.43–0.47	0.15	0.14–0.16
	100	0.23	0.19–0.26	0.04	0.04–0.06

For peer review only

STROBE Statement—Checklist of items that should be included in reports of ***cohort studies***

	Item No	Recommendation	Page No
**Title and abstract**	1	(*a*) Indicate the study's design with a commonly used term in the title or the abstract	1
		(*b*) Provide in the abstract an informative and balanced summary of what was done and what was found	2-3
**Introduction**			
Background/rationale	2	Explain the scientific background and rationale for the investigation being reported	4
Objectives	3	State specific objectives, including any prespecified hypotheses	5
**Methods**			
Study design	4	Present key elements of study design early in the paper	5-6
Setting	5	Describe the setting, locations, and relevant dates, including periods of recruitment, exposure, follow-up, and data collection	5-6
Participants	6	(*a*) Give the eligibility criteria, and the sources and methods of selection of participants. Describe methods of follow-up	5-6
		(*b*) For matched studies, give matching criteria and number of exposed and unexposed	
Variables	7	Clearly define all outcomes, exposures, predictors, potential confounders, and effect modifiers. Give diagnostic criteria, if applicable	6-7
Data sources/ measurement	8*	For each variable of interest, give sources of data and details of methods of assessment (measurement). Describe comparability of assessment methods if there is more than one group	6-7
Bias	9	Describe any efforts to address potential sources of bias	8-9
Study size	10	Explain how the study size was arrived at	5-6
Quantitative variables	11	Explain how quantitative variables were handled in the analyses. If applicable, describe which groupings were chosen and why	6-9
Statistical methods	12	(*a*) Describe all statistical methods, including those used to control for confounding	8-9
		(*b*) Describe any methods used to examine subgroups and interactions	9
		(*c*) Explain how missing data were addressed	5-6
		(*d*) If applicable, explain how loss to follow-up was addressed	8
		(*e*) Describe any sensitivity analyses	13
**Results**			
Participants	13*	(a) Report numbers of individuals at each stage of study—eg numbers potentially eligible, examined for eligibility, confirmed eligible, included in the study, completing follow-up, and analysed	9
		(b) Give reasons for non-participation at each stage	9
		(c) Consider use of a flow diagram	
Descriptive data	14*	(a) Give characteristics of study participants (eg demographic, clinical, social) and information on exposures and potential confounders	9
		(b) Indicate number of participants with missing data for each variable of interest	
		(c) Summarise follow-up time (eg, average and total amount)	
Outcome data	15*	Report numbers of outcome events or summary measures over time	9

For peer review only

For peer review only - http://bmjopen.bmj.com/site/about/guidelines.xhtml

Main results	16	(*a*) Give unadjusted estimates and, if applicable, confounder-adjusted estimates and their precision (eg, 95% confidence interval). Make clear which confounders were adjusted for and why they were included<br>(*b*) Report category boundaries when continuous variables were categorized<br>(*c*) If relevant, consider translating estimates of relative risk into absolute risk for a meaningful time period	9-11
Other analyses	17	Report other analyses done—eg analyses of subgroups and interactions, and sensitivity analyses	9
**Discussion**			
Key results	18	Summarise key results with reference to study objectives	11-12
Limitations	19	Discuss limitations of the study, taking into account sources of potential bias or imprecision. Discuss both direction and magnitude of any potential bias	13-14
Interpretation	20	Give a cautious overall interpretation of results considering objectives, limitations, multiplicity of analyses, results from similar studies, and other relevant evidence	11-13
Generalisability	21	Discuss the generalisability (external validity) of the study results	13
**Other information**			
Funding	22	Give the source of funding and the role of the funders for the present study and, if applicable, for the original study on which the present article is based	17

*Give information separately for exposed and unexposed groups.

Note: An Explanation and Elaboration article discusses each checklist item and gives methodological background and published examples of transparent reporting. The STROBE checklist is best used in conjunction with this article (freely available on the Web sites of PLoS Medicine at http://www.plosmedicine.org/, Annals of Internal Medicine at http://www.annals.org/, and Epidemiology at http://www.epidem.com/). Information on the STROBE Initiative is available at http://www.strobe-statement.org.

For peer review only

Midlife socioeconomic position and old-age dementia mortality: a large prospective register-based study from Finland

Journal:	*BMJ Open*
Manuscript ID	bmjopen-2019-033234.R2
Article Type:	Original research
Date Submitted by the Author:	13-Nov-2019
Complete List of Authors:	Korhonen, Kaarina; University of Helsinki, Population Research Unit<br>Einiö, Elina; University of Helsinki<br>Leinonen, Taina; Finnish Institute of Occupational Health,<br>Tarkiainen , Lasse ; University of Helsinki<br>Martikainen, Pekka; University of Helsinki
**Primary Subject Heading**:	Neurology
Secondary Subject Heading:	Public health, Sociology
Keywords:	Dementia < NEUROLOGY, Socioeconomic factors, Alzheimer's disease, Register study

Midlife socioeconomic position and old-age dementia mortality: a large prospective register-based study from Finland

Kaarina Korhonen[1], Elina Einiö[1,2,3], Taina Leinonen[4], Lasse Tarkiainen[1], Pekka Martikainen[1, 3, 5]

[1] Population Research Unit, Faculty of Social Sciences, University of Helsinki, Helsinki, Finland

[2] Department of Social Policy, London School of Economics and Political Science, London, United Kingdom

[3] Max Planck Institute for Demographic Research, Rostock, Germany

[4] Finnish Institute of Occupational Health, Helsinki, Finland

[5] Department of Public Health Sciences, Stockholm University, Stockholm, Sweden

Correspondence to:

Kaarina Korhonen

Faculty of Social Sciences

P.O. Box 18, 00014 University of Helsinki, Finland

+358 50 3199 388

kaarina.korhonen@helsinki.fi

Word count: 4117

For peer review only

For peer review only - http://bmjopen.bmj.com/site/about/guidelines.xhtml

Abstract

Objectives To assess the association between multiple indicators of socioeconomic position and dementia-related death, and to estimate the contribution of dementia to socioeconomic differences in overall mortality at older ages.

Design Prospective population-based register study.

Setting Finland.

Participants 11% random sample of the population aged 70–87 resident in Finland at the end of year 2000 (N=54 964).

Main outcome measure Incidence rates, Kaplan-Meier survival probabilities and Cox regression hazard ratios of dementia mortality in 2001–2016 by midlife education, occupational social class and household income measured at ages 53–57 years.

Results During the 528 387 person-years at risk, 11 395 individuals died from dementia (215.7 per 10 000 person-years). Lower midlife education, occupational social class and household income were associated with higher dementia mortality, and the differences persisted to the oldest old ages. Compared to mortality from all other causes, however, the socioeconomic differences emerged later. Dementia accounted for 28% of the difference between low and high education groups in overall mortality at age 70+, and for 21% of the difference between lowest and highest household income quintiles. All indicators of socioeconomic position were independently associated with dementia mortality, low household income being the strongest independent predictor (HR=1.24, 95% confidence interval 1.16–1.32), followed by basic education (HR=1.14, 1.06–1.23). Manual occupational social class was related to a 6% higher hazard (HR=1.06, 1.01–1.11) compared to non-manual social class. Adjustment for midlife economic activity, baseline marital status and chronic health conditions attenuated the excess hazard of low midlife household income, although significant effects remained.

For peer review only - http://bmjopen.bmj.com/site/about/guidelines.xhtml

Conclusion Several indicators of socioeconomic position predict dementia mortality independently and socioeconomic inequalities persist into the oldest old ages. The results demonstrate that dementia is among the most important contributors to socioeconomic inequalities in overall mortality at older ages.

Strengths and limitations of this study

- We used longitudinal registry data that permits a 15-year follow-up of dementia mortality with no attrition or recall bias.
- Dementia is documented in the national death register with high specificity.
- Due to the use of register data, traditional dementia risk factors such as smoking and physical activity could not be measured.
- All indicators of socioeconomic position were measured in midlife in order to avoid selection to socioeconomic groups on the basis of cognitive decline.
- This is the first study to show the contribution of dementia to the socioeconomic inequalities in overall mortality at older ages.

For peer review only

For peer review only - http://bmjopen.bmj.com/site/about/guidelines.xhtml

Introduction

Socioeconomic inequality in health and mortality is one of the most consistent findings in the demographic and social epidemiological literature. Lower education, occupational social class and income are strong predictors of all-cause and cause-specific mortality particularly among the working-age population, but inequalities are clear also at older ages.[1–4] Among the ageing population, the key factors affecting morbidity and disability are Alzheimer's disease and other forms of progressive dementia. Globally, an estimated 47 million people lived with dementia in 2015, and the number is projected to triple by 2050.[5] In England and Wales, dementia has already become the leading cause of death.[6] Despite the growing societal impact, however, no comprehensive understanding exists about the socioeconomic patterns of dementia mortality.

Educational inequalities in dementia mortality have previously been reported in studies following individuals from midlife or younger old ages[7,8] but not among the oldest old.[8,9] In a Norwegian health examination study, an educational pattern was present only among cohorts aged below 70 at baseline but not among those aged 70 and over.[8] Similarly, among a Finnish cohort aged 90 and over, no statistically significant educational gradient in dementia mortality emerged.[9] The lack of educational differentials among the oldest old may relate to selective survival. People with lower education experience higher mortality at younger ages, and those who survive to older ages do so because of their better health. Thus, the population surviving to older ages is more homogeneous in terms of health-related characteristics and, as a result, the socioeconomic differences in mortality are diminished. Another possible explanation for the lack of educational gradient in dementia mortality is the fact that the distribution of education in the oldest cohorts is highly skewed. Given that the majority of people in these cohorts have no more than basic education, other indicators of socioeconomic position (SEP) may be more suitable for identifying high-risk population subgroups.[2,10] Previous studies suggest that among adults in general, overall mortality disparities are greater or have increased to a greater extent in terms of occupational social class[11] and income[12,13] than education. Among the Finnish cohort of nonagenarians,[9] occupational social class

For peer review only - http://bmjopen.bmj.com/site/about/guidelines.xhtml

was a strong predictor of dementia mortality with a 3-fold hazard of dementia death among the unskilled manual workers compared to upper non-manuals. Personal income in midlife, however, was not related to dementia mortality among a cohort of Norwegian men.[14] To our knowledge, no previous study has assessed inequalities in dementia mortality by household income, a socioeconomic indicator that is more directly related to material resources available to the individual and that more rigorously captures the living conditions of the most disadvantaged population subgroups. A low household income may, in addition to material disadvantage, induce psychosocial stress, increasing the risk of dementia directly or through less favourable health behaviours. Disentangling the contributions of education, occupational social class and household income will thus provide important insights into the potential mechanisms how SEP shapes the risk of dementia death.

This study contributes to the existing knowledge by assessing socioeconomic inequalities in dementia mortality using multiple indicators of SEP, including education, occupational social class and household income. More specifically, the aims of the study were to 1) investigate the magnitude of socioeconomic inequalities in dementia mortality in relation to age, and compare the patterns to those in mortality from all other causes of death, 2) to quantify the contribution of dementia to the socioeconomic inequalities in overall mortality at older ages, and 3) to assess whether education, occupational social class and household income are independently related to dementia mortality once the other indicators are taken into account. This was because different indicators of SEP are correlated but each of them may have independent associations with dementia mortality. We further estimated models adjusting for confounders including marital status and chronic health conditions. We used longitudinal registry data on a large population-based sample, which permits a 15-year follow-up of dementia-related deaths with no attrition or recall bias. All indicators of SEP were measured in midlife in order to avoid selection to socioeconomic groups on the basis of cognitive decline.

Methods

Sample

For peer review only - http://bmjopen.bmj.com/site/about/guidelines.xhtml

For peer review only

We used an 11% random sample of the Finnish population in 1987–2007 drawn from the Statistics Finland population register, which covers all permanent residents. Statistics Finland linked the sample with information from various administrative registers including the national Death Register and healthcare registers using unique personal identification numbers assigned to all permanent residents.

In the present study, we included men and women aged 70–87 at the end of year 2000. For these cohorts, midlife socioeconomic characteristics could be identified using information from the Population Censuses conducted in 1970, 1975, 1980 and 1985. Individuals with missing census information due to residing outside of Finland (n=920) and those with missing household income information due to not being part of the household population in the census year (n=401) were excluded. 7 individuals emigrated during the first year of follow-up and thus were excluded from the analyses. The analytic sample consisted of 54 964 individuals.

Mortality data

Dates and causes of death were obtained from the Death Register. Dementia-related deaths were identified using the International Classification of Diseases 10th revision (ICD-10) codes F00–03 and G30 as the underlying or any of the three contributory causes of death reported on the death certificate. We identified 11 395 persons who died from dementia and 30 637 persons who died from other causes during the follow-up in 2001–2016.

Indicators of socioeconomic position

The information of all indicators of SEP was derived from the quinquennial population censuses of 1970–1985. A particular census year was chosen on the basis of the study subject's age so that the indicators were measured at around the age of 55 (range 53–57) for all. Education was indicated as the highest achieved qualification, categorised as tertiary (generally 13+ years of education; International Standard Classification of Education ISCED-1997 codes 5–6), secondary (10–12 years, ISCED 3–4), and basic education/no qualifications (9 years, ISCED 0–2). Occupational social class comprised five groups,

For peer review only - http://bmjopen.bmj.com/site/about/guidelines.xhtml

classified as non-manual, manual, self-employed farmer, other self-employed, and no occupation/unknown. Information of occupational social class in the census year was lacking for 10 465 individuals due to non-employment at that time. For 9942 individuals, the information could nevertheless be obtained from previous years in which the individuals were employed. Household income indicated the taxable annual income of all household members, including all income received in money or monetary benefit subject to tax. The information was obtained from the Finnish Tax Administration and the Social Insurance Institution of Finland. We adjusted for household composition using the OECD-modified equivalence scale.[15] Income quintiles were formed based on the household income distribution in the population aged 15 and over in the census year.

Covariates

The analyses incorporated information of economic activity measured from the census year because being out of the labour market may indicate poor health and affect dementia risk independently but also lead to reduced household income. Economic activity was classified as being in the labour force, retired and other inactive. Marital status was measured at baseline (the end of 2000), classified as married, divorced, widowed and never married. Baseline chronic health conditions included indicators of vascular and lifestyle risk factors for dementia,[16] and were identified from health registers in the five-year period before the baseline, covering 1996–2000. We used the diagnostic records of the hospital discharge register and patient censuses of the National Institute for Health and Welfare, and the records of prescription medicine purchases and of entitlement to special reimbursement for the medication expenses for certain chronic diseases maintained by the Social Insurance Institution of Finland. We included indicators for alcohol-related diseases and accidental poisoning by alcohol, asthma and other chronic obstructive pulmonary disease (COPD), diabetes and heart disease (for coding see Supplementary Table 1). These chronic conditions may confound the association between midlife SEP and dementia mortality as the diseases usually develop over a long period of time and thus reflect health behaviours or health problems already present in midlife. To account for potential regional variance in socioeconomic characteristics and mortality,

For peer review only - http://bmjopen.bmj.com/site/about/guidelines.xhtml

we included dummies for region of residence (Western Finland, Helsinki capital region, rest of Southern Finland, Eastern Finland, and Lapland) and the degree of urbanisation of the municipality of residence, a variable based on the proportion of population living in urban settlements and the population of the largest urban settlement in the municipality (urban, semi-urban and rural).

Statistical analyses

We followed the study population for dementia mortality from 1 January 2001 until 31 December 2016. Individuals were censored on the date of death, at the end of the year preceding emigration, or at the end of 2016, whichever came first.

For descriptive statistics, we calculated age-adjusted dementia mortality rates per 10 000 person-years at risk by indicators of SEP and the covariates. In order to assess the magnitude of socioeconomic inequalities in relation to age, we estimated Kaplan–Meier survival functions by education, occupational social class and household income. In these analyses, we contrasted the survival functions of the highest and lowest education groups, non-manual and manual employees and the highest and lowest household income quintiles. The equality of survival functions was tested using log-rank tests. For the comparison between dementia mortality and the more general mortality patterns, separate Kaplan-Meier survival functions were estimated for mortality from all other causes of death. We also estimated hazard ratios and their 95% confidence intervals for low versus high socioeconomic groups at the age of 70–79, 80–89 and 90 years and over.

To quantify the contribution of dementia to socioeconomic differences in overall mortality at older ages, we calculated absolute rate differences in mortality between socioeconomic groups (basic vs. tertiary education, manual vs. non-manual occupational social class, lowest vs. highest household income quintile) by cause of death. The contribution was determined by the rate difference in dementia mortality as a percentage of the rate difference in total mortality. Because the level of dementia mortality increases substantially with age, we also assessed age-specific contributions (at the age of 70–79, 80–89 and 90+).

For peer review only - http://bmjopen.bmj.com/site/about/guidelines.xhtml

To estimate the independent associations between each indicator of SEP and dementia mortality, we used Cox regression models. Attained age in years was used as the time scale, and thus all analyses adjusted for the confounding effect of age.[17] We first estimated crude associations between each indicator and dementia mortality, adjusting for calendar year dummies, gender, region of residence and the degree of urbanisation (model 1). Model 2 included education, occupational social class and household income as covariates, thus showing mutually adjusted associations. Midlife economic activity was adjusted for in model 3. We further adjusted for baseline marital status and chronic health conditions in model 4 to assess the extent to which these confounding factors attenuated the relative hazard attached to each socioeconomic indicator.

We tested for interactions between gender and each socioeconomic indicator using likelihood ratio test. Interactions were statistically nonsignificant ($p>0.05$), and thus we conducted all analyses for men and women combined. We also tested for interactions of all pairwise combinations of the socioeconomic indicators, adjusting for the covariates of model 1. These interactions were all statistically nonsignificant ($p>0.05$). All analyses were performed using Stata 15.1.[18]

Patient and public involvement

No patients were involved in setting the research question or the outcome measures, nor were they involved in developing plans for design or implementation of the study. No patients were asked to advise on interpretation or writing up of results. There are no plans to disseminate the results of the research to study participants or the relevant patient community.

Results

Table 1 shows the distribution of the study population by indicators of midlife SEP, economic activity and baseline characteristics. The vast majority of individuals (77.2%) had no higher than basic education, and manual employees formed the largest occupational social class (43.6%). Higher household income quintiles were over-represented among the study population due to the higher incomes of the middle aged compared

For peer review only - http://bmjopen.bmj.com/site/about/guidelines.xhtml

to the rest of the population and also partly because of greater mortality of the lower income groups between the time of measurement of midlife income and the baseline. During the 528 387 person-years at risk 11 395 individuals died from dementia, the average age-adjusted dementia mortality rate being 223.1 and 210.8 per 10 000 person-years among men and women, respectively. The rate was higher for those with lower education, occupational social class and household income, and also for the non-married and people with chronic health conditions apart from asthma and other COPD.

Kaplan–Meier survival functions in Figure 1 show that dementia mortality differed by all indicators of SEP (log rank test, $p<0.001$ for each indicator), and that the age patterns differed between the indicators (for 95% confidence intervals see Supplementary Table 2). The inequalities emerged at an earlier age when SEP was measured in terms of household income (Panel c) compared to education (Panel a) and occupational social class (Panel b). At the age of 90 years and above, by contrast, the differences were more pronounced when SEP was measured in terms of education. Nevertheless, inequalities in dementia mortality emerged substantially later in life compared to mortality from all other causes. Hazard ratios in Table 2 show that relative inequalities in mortality tended to diminish with age for all indicators of SEP regardless of cause of death. However, education differences in dementia mortality showed a different age pattern in that the point estimates indicated stable inequality with age.

Overall, dementia contributed to 28.1% of educational and 20.9% of household income differences in total mortality at the age of 70 and over (Table 2). The contribution to occupational social class differences was somewhat smaller (16.7%). The contribution of dementia to socioeconomic inequalities substantially increased from the age of 70–79 to 90 years and over.

Cox regression models in Table 3 show adjusted hazard ratios (HR) for dementia mortality across all ages from 70 years and over. Adjusted for calendar year, gender, region of residence and the degree of urbanisation in model 1, the associations were strongest for basic education (HR=1.23, 95% CI 1.15–1.32), unknown occupational social class (HR=1.20, 1.00–1.44), and the lowest household income quintile

For peer review only - http://bmjopen.bmj.com/site/about/guidelines.xhtml

(HR=1.28, 1.20–1.35). Mutual adjustment of socioeconomic indicators in model 2 attenuated educational differences by about 40%, and unknown occupational social class to a non-significant level. Basic education (HR=1.14, 1.06–1.23), manual occupational social class (HR=1.06, 1.01–1.11) and three lowest household income quintiles (for the lowest quintile HR=1.24, 1.16–1.32) all predicted dementia mortality independently of each other. Adjustment for midlife economic activity in model 3 attenuated the excess hazard particularly of the lower household income quintiles. Adjustment for baseline marital status and chronic health conditions in model 4 contributed to a small change in the estimates, the attenuation being largest for the lowest household income quintile. In this full model, basic education increased the hazard of dementia death by 14% (1.06–1.23), manual occupational social class by 5% (1.00–1.10) and the two lowest household income quintiles by 7–13% (HR=1.07, 1.00–1.14 to HR=1.13, 1.06–1.22).

Discussion

Main findings and their interpretation

In this study we have shown that dementia mortality at older ages is socioeconomically patterned in terms of multiple indicators of SEP. People with lower education, occupational social class and household income have a higher risk of dementia death compared to those with higher SEP. These results add to the literature on socioeconomic inequalities in old-age mortality, which has previously shown a socioeconomic pattern in many other specific causes of death such as cardiovascular diseases, COPD and cancer.[1] Our results indicate, moreover, that dementia is an important factor in overall socioeconomic inequalities in old-age mortality, contributing to 21–28% of household income and educational differences in total mortality among the population aged 70 and over. The contribution of dementia to overall socioeconomic inequalities in mortality increased substantially with age, which relates to the increasing proportion of deaths attributable to dementia with advancing age.[19]

A major difference in the patterns between dementia mortality and mortality from all other causes of death was that socioeconomic inequalities in dementia mortality emerged later and the inequalities in dementia

For peer review only - http://bmjopen.bmj.com/site/about/guidelines.xhtml

For peer review only

mortality between high and low education groups persisted in the same magnitude to the oldest old ages (90 years and above). By contrast, inequalities in mortality from other causes of death tended to diminish with age. The attenuation of socioeconomic inequalities with age is a general finding,[1,2] and may partly relate to selective survival, suggesting that people who survive to very old age have more similar health profiles across socioeconomic groups. Our results show, however, that even among people who survive to the oldest old age, education groups differ in neurological health. This is a novel finding in that previous studies have identified consistent socioeconomic inequalities in dementia mortality only among the younger old[7,8] but the results have been mixed for the oldest old.[8,9] Participation bias may at least partly explain the differences in findings; people of older age, lower SEP and with health problems are less likely to participate in surveys and studies involving health examinations. Our study employed register data on a population-based cohort and thus is not affected by participation or attrition biases.

The age patterns in dementia mortality differed between indicators of SEP: while educational differences were more pronounced among the oldest old (90 years and over), the differences among the younger old (70–79 years) were largest when SEP was measured in terms of household income. Individuals in the lowest income quintile represent the most disadvantaged population subgroups with multiple potential dementia risk factors. Our findings show that the higher dementia mortality of the lowest household income quintiles was strongly — although not fully — confounded by greater morbidity of these groups. Severe health problems that were already present in midlife have potentially affected both household incomes and the risk of dementia death. However, we cannot rule out the possibility of mediation, especially because chronic health conditions were measured after midlife income; impoverished material conditions may also affect dementia risk through, for example, health-related behaviours, cardiovascular risk factors[16] and psychological stress.[20] In the presence of mediation, our estimates would be conservative as they would overadjust part of the effect of socioeconomic disadvantage. Future studies are needed to establish the causal relationship between mediating factors and dementia mortality using mediation analysis techniques.

For peer review only - http://bmjopen.bmj.com/site/about/guidelines.xhtml

Education, in turn, may have particular benefits above and beyond physical health factors among the population surviving to the oldest old age. Our results show persistent educational differences in dementia mortality, and the association was not confounded by chronic health conditions, economic activity or marital status. Education is a well-established predictor of dementia incidence,[21] although the exact mechanisms are still not known. Brain autopsy studies indicate, in line with the cognitive reserve hypothesis,[22] that education is not associated with the burden of neuropathology at death but higher education enables individuals to compensate longer for the neuropathological changes before developing clinical symptoms of dementia.[23] Thus, it is possible that the educational differences in dementia mortality we found in our study are due to competing risks; people with higher education died from other causes before they reached the phase of clinical dementia or died from other causes before dementia progressed to death. However, the empirical evidence for the cognitive reserve hypothesis remains open to debate. For example, several studies have not identified educational differences in survival time after dementia onset,[24] which is among the key hypotheses in the cognitive reserve model.[25] Therefore, it is plausible that higher education enhances brain health and protects against (or postpones) not only the clinical symptoms but also the development of neurodegenerative disorders.

Occupational social class differences in dementia mortality were modest following adjustment for education and household income. In particular, the high hazard among those with no occupation disappeared after these adjustments indicating that this group experienced multiple socioeconomic disadvantages. The results suggest, nevertheless, that higher social class occupations may involve greater cognitive demands and intellectual engagement, and thus enhance cognitive health.[26,27] In contrast, lower class occupations or long periods of economic inactivity due to unemployment or early retirement may reduce opportunities for cognitive investment. Overall, the results of this study suggest that all three indicators of SEP are important factors in bringing about socioeconomic differences in dementia mortality, also influencing inequalities in overall mortality among the older population.

Methodological considerations

For peer review only - http://bmjopen.bmj.com/site/about/guidelines.xhtml

We used a unique population-representative sample of older adults in Finland with 11 395 dementia deaths identified from the National Death Register. The register-based sample was not affected by participation or attrition bias, which are common limitations of many cohort designs, particularly among the older population. The population register encompasses rich information on demographic and socioeconomic characteristics of individuals over the life course, and is not subject to bias from individuals' self-reports or recollection.

Despite the rich register data, our study also has some limitations. First, we could only identify dementia cases that have been recorded on the death certificate. According to a validation study for identifying dementia in the Finnish national registers, the documentation of dementia as the cause of death has improved since the late 1990s, and the specificity is particularly high.[28] To minimise any bias arising from potential underreporting of dementia as the underlying cause of death, we applied the multiple-cause approach and included also cases where dementia was recorded as any of the three contributory causes.[29] Defined this way, we identified 21% of all deaths at the age of 70 and over to be attributable to dementia. This relatively high proportion is in line with that reported in England and Wales, where dementia accounted for 19% of all deaths at the age of 80 and over.[30] Furthermore, we ran sensitivity analyses with interaction with calendar year, and found that the associations between the indicators of SEP and dementia mortality did not vary in time. Therefore, we believe our results are not biased by overreporting or underreporting of dementia as the cause of death or by changes in documentation practices.

Second, the information of household income was based on taxable income and the variable thus excludes certain monetary transfers such as housing allowance and social assistance. These means-tested sources of income may be especially relevant for people with health problems and those outside the labour market. This might lead to overestimation of the income effect. Information of disposable income was not available for years 1970–1985, but we carried out a robustness check for the correlation between taxable and disposable household incomes (as continuous variables) using the population aged 15 and over in 1995 and found the correlation to be as high as 0.97 (among the population aged 53–57 in 1995 the correlation was

For peer review only - http://bmjopen.bmj.com/site/about/guidelines.xhtml

0.98). Therefore, it is unlikely that the use of disposable income would change the ranking of individuals in the household income distribution to the extent that it would affect our main findings.

Finally, the causal relationship between SEP and dementia is difficult to establish in observational studies. We therefore measured all socioeconomic characteristics 15–30 years before the mortality follow-up, and it is thus very unlikely that any symptoms of dementia affected the midlife socioeconomic attainment of individuals. Nevertheless, we cannot exclude the possibility that early cognitive decline may have affected midlife SEP, especially measured in term of occupational social class and household income. Also, given the small proportion of people with tertiary education in these cohorts (10%), it is possible that this forms a select group with multiple advantages including higher childhood SEP and early cognitive ability. Because register data does not cover information of traditional risk factors related to health behaviours such as smoking and physical activity, we included indicators of chronic conditions to measure cardiovascular and life style risk factors for dementia that may confound the association between SEP and dementia mortality.

Conclusions

This study provides new insight into the socioeconomic inequalities in old-age mortality by showing a consistent pattern in dementia mortality by multiple indicators of SEP. Low education, occupational social class and household income were all associated with higher risk of dementia death, although the socioeconomic differences emerged later than in mortality from other causes. Household income differences in dementia mortality were more pronounced among the younger old, and the associations were largely attributable to other chronic health conditions such as diabetes and alcohol-related diseases. Educational inequalities, by contrast, were independent of chronic health conditions and became more pronounced at the oldest old age where mortality inequalities generally begin to attenuate. The results indicate that dementia mortality may be amenable to socioeconomic interventions in midlife. The findings also suggest that dementia contributes to socioeconomic inequalities in overall mortality at older ages and,

For peer review only - http://bmjopen.bmj.com/site/about/guidelines.xhtml

thus, dementia prevention is important from the point of view of socioeconomic inequalities in total mortality.

For peer review only

For peer review only - http://bmjopen.bmj.com/site/about/guidelines.xhtml

REFERENCES

1 Huisman M, Kunst AE, Bopp M, *et al.* Educational inequalities in cause-specific mortality in middle-aged and older men and women in eight western European populations. *The Lancet* 2005;**365**:493–500.

2 Huisman M, Read S, Towriss CA, *et al.* Socioeconomic Inequalities in Mortality Rates in Old Age in the World Health Organization Europe Region. *Epidemiol Rev* 2013;**35**:84–97.

3 Marmot MG, Shipley MJ. Do socioeconomic differences in mortality persist after retirement? 25 Year follow up of civil servants from the first Whitehall study. *BMJ* 1996;**313**:1177–80.

4 Demakakos P, Biddulph JP, Bobak M, *et al.* Wealth and mortality at older ages: a prospective cohort study. *J Epidemiol Community Health* 2016;**70**:346–353.

5 Prince M, Wimo A, Guerchet M, *et al.* World Alzheimer Report 2015—The Global Impact of Dementia: an analysis of prevalence, incidence, cost and trends. 2015. Available: https://www.alz.co.uk/research/WorldAlzheimerReport2015.pdf (accessed 13 Sep 2017).

6 Office for National Statistics. Deaths registered in England and Wales (series DR): 2016. 2017. Available:https://www.ons.gov.uk/peoplepopulationandcommunity/birthsdeathsandmarriages/deaths/bulletins/deathsregisteredinenglandandwalesseriesdr/2016#dementia-and-alzheimer-disease-remained-the-leading-cause-of-death-in-2016 (accessed 20 Sep 2019).

7 Russ TC, Stamatakis E, Hamer M, *et al.* Socioeconomic status as a risk factor for dementia death: individual participant meta-analysis of 86 508 men and women from the UK. *The British journal of psychiatry* 2013;**203**:10–17.

8 Strand BH, Langballe EM, Rosness TA, *et al.* Age, education and dementia related deaths. The Norwegian Counties Study and the Cohort of Norway. *Journal of the neurological sciences* 2014;**345**:75–82.

9 Enroth L, Raitanen J, Hervonen A, *et al.* Is socioeconomic status a predictor of mortality in nonagenarians? The vitality 90+ study. *Age and ageing* 2014;**44**:123–129.

10 Martelin T. Mortality by indicators of socioeconomic status among the Finnish elderly. *Social science & medicine* 1994;**38**:1257–1278.

11 Martikainen P, Blomgren J, Valkonen T. Change in the total and independent effects of education and occupational social class on mortality: analyses of all Finnish men and women in the period 1971–2000. *Journal of Epidemiology & Community Health* 2007;**61**:499–505.

12 Rognerud MA, Zahl P-H. Social inequalities in mortality: changes in the relative importance of income, education and household size over a 27-year period. *The European Journal of Public Health* 2005;**16**:62–68.

13 Geyer S, Hemström Ö, Peter R, *et al.* Education, income, and occupational class cannot be used interchangeably in social epidemiology. Empirical evidence against a common practice. *Journal of Epidemiology & Community Health* 2006;**60**:804–810.

14 Strand BH, Skirbekk V, Rosness TA, *et al.* Income in midlife and dementia related mortality over three decades: A Norwegian prospective study. *eNeurologicalSci* 2015;**1**:24–29.

For peer review only - http://bmjopen.bmj.com/site/about/guidelines.xhtml

15 OECD. *OECD Framework for Statistics on the Distribution of Household Income, Consumption and Wealth*. Paris: OECD Publishing 2013. Available: http://dx.doi.org/10.1787/9789264194830-en (accessed 3 May 2018).

16 Baumgart M, Snyder HM, Carrillo MC, *et al.* Summary of the evidence on modifiable risk factors for cognitive decline and dementia: a population-based perspective. *Alzheimer's & dementia: the journal of the Alzheimer's Association* 2015;**11**:718–726.

17 Lamarca R, Alonso J, Gómez G, *et al.* Left-truncated data with age as time scale: an alternative for survival analysis in the elderly population. *The Journals of Gerontology Series A: Biological Sciences and Medical Sciences* 1998;**53**:M337–M343.

18 StataCorp. Stata Statistical Software: Release 15. College Station, TX: StataCorp LLC. 2017.

19 Knapp M, Prince M, Albanese E, *et al. Dementia UK: The full report*. London: Alzheimer's Society 2007.

20 Johansson L, Guo X, Waern M, *et al.* Midlife psychological stress and risk of dementia: a 35-year longitudinal population study. *Brain* 2010;**133**:2217–2224.

21 Livingston G, Sommerlad A, Orgeta V, *et al.* Dementia prevention, intervention, and care. *The Lancet* Published Online First: 2017. Available: https://tbhtoolkits.com/media/Dementia-Prevention-Intervention-and-Care-Lancet-2017-1.pdf (accessed 11 Sep 2017).

22 Stern Y. What is cognitive reserve? Theory and research application of the reserve concept. *Journal of the International Neuropsychological Society* 2002;**8**:448–460.

23 Brayne C, Ince PG, Keage HA, *et al.* Education, the brain and dementia: neuroprotection or compensation? EClipSE Collaborative Members. *Brain* 2010;**133**:2210–2216.

24 Paradise M, Cooper C, Livingston G. Systematic review of the effect of education on survival in Alzheimer's disease. *International psychogeriatrics* 2009;**21**:25–32.

25 Stern Y, Tang MX, Denaro J, *et al.* Increased risk of mortality in Alzheimer's disease patients with more advanced educational and occupational attainment. *Annals of Neurology: Official Journal of the American Neurological Association and the Child Neurology Society* 1995;**37**:590–595.

26 Kröger E, Andel R, Lindsay J, *et al.* Is complexity of work associated with risk of dementia? The Canadian Study of Health and Aging. *American journal of epidemiology* 2008;**167**:820–830.

27 Karp A, Andel R, Parker MG, *et al.* Mentally stimulating activities at work during midlife and dementia risk after age 75: follow-up study from the Kungsholmen Project. *The American Journal of Geriatric Psychiatry* 2009;**17**:227–236.

28 Solomon A, Ngandu T, Soininen H, *et al.* Validity of dementia and Alzheimer's disease diagnoses in Finnish national registers. *Alzheimer's & Dementia* 2014;**10**:303–309. doi:10.1016/j.jalz.2013.03.004

29 Garcia-Ptacek S, Kåreholt I, Cermakova P, *et al.* Causes of Death According to Death Certificates in Individuals with Dementia: A Cohort from the Swedish Dementia Registry. *Journal of the American Geriatrics Society* 2016;**64**. doi:10.1111/jgs.14421

For peer review only

For peer review only - http://bmjopen.bmj.com/site/about/guidelines.xhtml

30 Office for National Statistcs. Deaths registered in England and Wales, 2016 (dataset). 2017. Available:https://www.ons.gov.uk/file?uri=/peoplepopulationandcommunity/birthsdeathsandmarriages/deaths/datasets/deathsregisteredinenglandandwalesseriesdrreferencetables/2016/drtables16.xls (accessed 20 Sep 2019).

For peer review only

For peer review only - http://bmjopen.bmj.com/site/about/guidelines.xhtml

Footnotes

Contributors: KK, EE, TL, LT and PM participated in designing the study, generating hypotheses, interpreting the data and critically revised the manuscript for important intellectual content. KK analysed the data, conducted the literature review and wrote the first draft of the manuscript.

Funding: KK was supported by the Eino Jutikkala Fund. PM was supported by the Academy of Finland, and the Strategic Research Council PROMEQ project (#303615). PM was also supported by the European Union Horizon2020 Programme under grant agreement n° 667661 (Promoting mental wellbeing in the ageing population - MINDMAP). The study does not necessarily reflect the Commission's views and in no way anticipates the Commission's future policy in this area. The funders had no role in study design, data collection and analysis, decision to publish, or preparation of the manuscript.

Competing interests: None declared.

Data sharing statement: Statistics Finland, the National Institute for Health and Welfare and the Social Insurance Institution of Finland have collected and own the data. Due to data protection regulations, the authors are not allowed to make the data available to third parties. Researchers can apply for data access by contacting the register-holding institutions: Statistics Finland (http://stat.fi/index_en.html); National Institute for Health and Welfare (https://thl.fi/en); Social Insurance Institution of Finland (https://www.kela.fi/web/en).

Ethics approval: The study has been approved by Statistics Finland Board of Ethics (permit TK-53-339-13). The data were collected for routine administrative registration purposes and, therefore, informed consent of the participants was not obtained. These register data can be used for scientific purposes under the Personal Data Act and the Statistics Act. Statistics Finland anonymised the data prior to providing them to researchers.

For peer review only - http://bmjopen.bmj.com/site/about/guidelines.xhtml

Table 1. Distribution of the study population, dementia deaths and age-adjusted dementia mortality rates (per 10 000 person-years) by indicators of midlife socioeconomic position and economic activity and baseline characteristics, Finnish men and women in 2001–2016

			Dementia deaths		
	N	%	n	Rate	95% CI
Mean age at baseline (SD)	76.4 (4.8)				
Gender					
Men	20100	36.6	3409	223.1	215.6–230.5
Women	34864	63.4	7986	210.8	206.3–215.4
Education[a]					
Tertiary	5445	9.9	1014	185.5	174.3–196.7
Secondary	7074	12.9	1446	205.7	195.4–216.1
Basic	42445	77.2	8936	221.8	217.3–226.3
Occupational social class[a]					
Non-manual	17015	31.0	3524	201.1	194.6–207.6
Manual	23951	43.6	4882	228.4	222.1–234.6
Self-employed farmer	10204	18.6	2211	215.7	206.9–224.6
Other self-employed	3271	6.0	657	212.2	196.3–228.1
No occupation/unknown	523	1.0	121	239.2	196.5–282.0
Household income[a]					
Highest quintile	13667	24.9	2715	196.9	189.7–204.2
2nd	10522	19.1	2098	209.6	200.9–218.4
3rd	10110	18.4	2114	217.3	208.2–226.3
4th	10292	18.7	2183	223.2	214.0–232.3
Lowest quintile	10373	18.9	2285	241.5	231.8–251.2
Economic activity[a]					
Active	37266	67.8	7585	208.1	203.6–212.7
Retired	8881	16.2	1742	257.1	245.2–269.0
Other inactive	8817	16.0	2068	212.7	203.7–221.8
Marital status					
Married	24789	45.1	4471	208.7	202.6–214.8
Divorced	4056	7.4	797	237.0	220.9–253.2
Widowed	20997	38.2	5000	214.3	208.4–220.3
Never married	5122	9.3	1127	240.9	227.2–254.7
Chronic health conditions					
Alcohol-related diseases	308	0.6	68	505.3	381.2–629.3
Asthma and COPD	4510	8.2	789	232.9	216.9–248.9
Diabetes	6714	12.2	1240	275.0	259.8–290.2

For peer review only - http://bmjopen.bmj.com/site/about/guidelines.xhtml

Heart disease	18094	32.9	3562	237.1	229.5–244.7
Region of residence					
Western Finland	25078	45.6	4979	204.1	198.6–209.7
Helsinki capital region	7449	13.6	1582	208.3	198.3–218.4
Rest of Southern Finland	12056	21.9	2464	214.8	206.5–223.0
Eastern Finland	8458	15.4	1916	250.1	239.2–261.0
Lapland	1923	3.5	454	261.5	238.1–285.0
Degree of urbanisation					
Urban	29853	51.0	6401	217.2	212.0–222.4
Semi-urban	9285	17.7	1831	210.6	201.2–220.0
Rural	15826	31.3	3163	215.3	208.0–222.7
Total	54964	100.0	11395	215.7	211.7–219.7

Abbreviations: CI, confidence intervals; COPD, chronic obstructive pulmonary diseases

[a] Information from the population censuses of 1970–1985, the study population being aged 53–57 years

For peer review only

Table 2. Relative and absolute differences in mortality between high and low socioeconomic groups[a] by cause of death and age, and contribution (%) of dementia and other causes of death to socioeconomic differences in total mortality by age, Finnish men and women in 2001–2016

	70–79 years				80–89 years				90+ years				All ages 70+
	HR	95% CI	Rate difference	Contribution (%)	HR	95% CI	Rate difference	Contribution (%)	HR	95% CI	Rate difference	Contribution (%)	Contribution (%)
Education[b]													
Dementia	1.24	0.97–1.58	8.7	8.3	1.19	1.09–1.29	40.4	33.5	1.24	1.10–1.40	157.9	51.8	28.1
Other causes	1.38	1.26–1.50	96.1	91.7	1.11	1.06–1.17	83.0	68.9	1.13	1.03–1.24	146.9	48.2	71.8
Total mortality	1.36	1.25–1.48	104.8	100.0	1.13	1.09–1.18	120.4	102.4	1.17	1.09–1.26	304.8	100.0	100.0
Occupational social class[c]													
Dementia	1.22	1.03–1.44	7.7	6.3	1.17	1.10–1.23	35.3	20.3	1.09	1.00–1.17	63.5	22.8	16.7
Other causes	1.44	1.36–1.53	114.2	93.8	1.24	1.20–1.29	138.7	79.7	1.19	1.12–1.27	215.0	77.2	83.3
Total mortality	1.41	1.34–1.49	121.8	100.1	1.22	1.19–1.26	173.9	100.0	1.15	1.09–1.21	278.4	100.0	100.0
Household income[d]													
Dementia	1.63	1.32–2.01	22.4	12.9	1.22	1.14–1.31	46.3	21.4	1.19	1.08–1.32	135.6	35.2	20.9
Other causes	1.54	1.43–1.66	151.0	87.1	1.24	1.19–1.30	169.7	78.6	1.19	1.10–1.28	249.1	64.8	79.2
Total mortality	1.55	1.44–1.67	173.3	100.0	1.24	1.19–1.29	216.0	100.0	1.19	1.12–1.26	384.7	100.0	100.0

Abbreviations: CI, confidence interval; HR, hazard ratio
Hazard ratios adjusted for calendar year. Age-adjusted incidence rates calculated as dementia deaths per 10,000 person-years at risk. Contribution of dementia determined by the rate difference in dementia mortality as a percentage of the rate difference in total mortality

[a] Information from the population censuses of 1970–1985, the study population being aged 53–57 years

[b] Tertiary vs. basic education

[c] Non-manual vs. manual occupational social class

[d] Highest vs. lowest household income quintiles

Table 3. Hazard ratios and 95% confidence intervals for dementia mortality by indicators of midlife socioeconomic position[a], Finnish men and women in 2001–2016, n=54,964

Indicator of socioeconomic position	Model 1[b]		Model 2[c]		Model 3[d]		Model 4[e]	
	HR	95% CI	HR	95% CI	HR	95% CI	HR	95% CI
Education								
Tertiary	1.00		1.00		1.00		1.00	
Secondary	1.14	1.05–1.23	1.08	0.99–1.17	1.08	0.99–1.17	1.08	0.99–1.18
Basic	1.23	1.15–1.32	1.14	1.06–1.23	1.14	1.05–1.22	1.14	1.06–1.23
Occupational social class								
Non-manual	1.00		1.00		1.00		1.00	
Manual	1.14	1.09–1.20	1.06	1.01–1.11	1.05	1.00–1.11	1.05	1.00–1.10
Farmer	1.08	1.02–1.15	0.96	0.90–1.03	0.97	0.91–1.04	0.98	0.92–1.05
Other self-employed	1.05	0.96–1.14	0.98	0.90–1.07	0.99	0.91–1.08	1.00	0.92–1.09
No occupation/unknown	1.20	1.00–1.44	1.04	0.87–1.25	0.94	0.78–1.14	0.94	0.78–1.13
Household income								
Highest quintile	1.00		1.00		1.00		1.00	
2nd	1.08	1.02–1.14	1.04	0.98–1.10	1.03	0.98–1.10	1.02	0.96–1.09
3rd	1.13	1.07–1.20	1.08	1.02–1.15	1.07	1.00–1.14	1.05	0.99–1.12
4th	1.17	1.10–1.24	1.13	1.06–1.20	1.10	1.03–1.17	1.07	1.00–1.14
Lowest quintile	1.28	1.20–1.35	1.24	1.16–1.32	1.18	1.10–1.26	1.13	1.06–1.22

Abbreviations: CI, confidence interval; HR, hazard ratio

All models used age as time scale and adjusted for calendar year, gender, region of residence and the degree of urbanisation

[a] Information from the population censuses of 1970–1985, the study population being aged 53–57 years

[b] Model 1: each indicator of socioeconomic position separately

[c] Model 2: indicators of socioeconomic position mutually adjusted

[d] Model 3: model 2 + midlife economic activity

[e] Model 4: model 3 + baseline marital status and chronic health conditions (alcohol-related diseases, asthma and chronic obstructive pulmonary disease, diabetes and heart disease)

For peer review only - http://bmjopen.bmj.com/site/about/guidelines.xhtml

Figure 1. Kaplan–Meier survival probabilities for dementia mortality and mortality from all other causes of death by a) education, b) occupational social class and c) household income quintile (Q1=highest, Q5=lowest), Finnish men and women in 2001–2016. Information of midlife socioeconomic position obtained from the population censuses of 1970–1985, the study population being aged 53–57 years

For peer review only

For peer review only - http://bmjopen.bmj.com/site/about/guidelines.xhtml

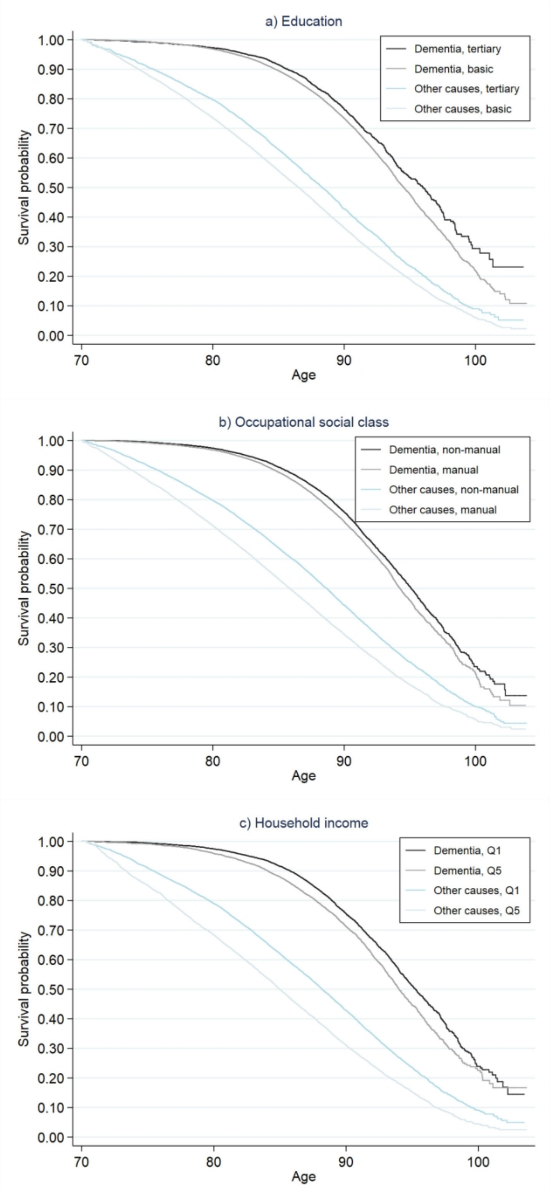

Figure 1. Kaplan–Meier survival probabilities for dementia mortality and mortality from all other causes of death by a) education, b) occupational social class and c) household income quintile (Q1=highest, Q5=lowest), Finnish men and women in 2001–2016. Information of midlife socioeconomic position obtained from the population censuses of 1970–1985, the study population being aged 53–57 years

122x265mm (300 x 300 DPI)

Supplementary Table 1. Classification of chronic health conditions used as covariates in the study

Condition	Hospital diagnoses (ICD-10)	Prescription medication (ATC)	Special reimbursement category (Finnish disease code)
Alcohol-related diseases and accidental poisoning by alcohol	F10–19, G31.2, G40.51, G62.1, G72.1, I42.6, K29.2, K70, K86.0, O35.4, X45		
Asthma and other COPD	J43–46		203
Diabetes	E10–14	A10	103
Heart disease	I00–09, I20–52		201, 206, 207

Abbreviations: ATC, Anatomical Therapeutic Chemical; COPD, chronic obstructive pulmonary diseases; ICD, International Classification of Diseases

review only

Supplementary Table 2. Kaplan-Meier survival probabilities at specific ages by midlife a) education, b) occupational social class and c) household income quintile (Q1=highest, Q5=lowest), Finnish men and women in 2001–2016

		Dementia		Other causes	
	Age	Survivor function	95% CI	Survivor function	95% CI
a) Education					
Tertiary	70	1.00		1.00	
	75	0.99	0.99–1.00	0.91	0.89–0.92
	80	0.97	0.97–0.98	0.80	0.78–0.82
	85	0.92	0.90–0.92	0.63	0.61–0.65
	90	0.76	0.75–0.78	0.43	0.41–0.44
	95	0.53	0.50–0.56	0.23	0.22–0.25
	100	0.29	0.24–0.35	0.09	0.07–0.11
Basic	70	1.00		1.00	
	75	0.99	0.99–1.00	0.88	0.87–0.89
	80	0.97	0.97–0.97	0.74	0.73–0.74
	85	0.89	0.89–0.90	0.56	0.55–0.56
	90	0.73	0.73–0.74	0.36	0.36–0.37
	95	0.48	0.47–0.49	0.19	0.18–0.20
	100	0.22	0.20–0.24	0.06	0.05–0.07
b) Occupational social class					
Non-manual	70	1.00		1.00	
	75	1.00	0.99–1.00	0.92	0.90–0.93
	80	0.97	0.97–0.98	0.80	0.79–0.81
	85	0.91	0.90–0.92	0.64	0.63–0.65
	90	0.76	0.75–0.77	0.44	0.43–0.45
	95	0.51	0.49–0.52	0.25	0.24–0.26
	100	0.24	0.21–0.27	0.10	0.09–0.11
Manual	70	1.00		1.00	
	75	0.99	0.99–0.99	0.87	0.85–0.88
	80	0.97	0.96–0.97	0.71	0.70–0.72
	85	0.89	0.89–0.90	0.53	0.52–0.54
	90	0.73	0.72–0.73	0.34	0.34–0.35
	95	0.46	0.45–0.47	0.17	0.17–0.18
	100	0.21	0.18–0.24	0.06	0.05–0.07

	Age	Dementia		Other causes	
		Survivor function	95% CI	Survivor function	95% CI
c) Household income					
Q1	70	1.00		1.00	
	75	0.99	0.99–1.00	0.91	0.90–0.92
	80	0.97	0.97–0.98	0.79	0.78–0.80
	85	0.92	0.91–0.92	0.62	0.61–0.63
	90	0.75	0.74–0.76	0.43	0.42–0.44
	95	0.51	0.49–0.53	0.24	0.22–0.25
	100	0.24	0.20–0.28	0.09	0.08–0.11
Q5	70	1.00		1.00	
	75	0.99	0.99–1.00	0.85	0.83–0.87
	80	0.96	0.95–0.97	0.68	0.67–0.70
	85	0.88	0.87–0.89	0.50	0.48–0.51
	90	0.71	0.70–0.72	0.31	0.30–0.32
	95	0.45	0.43–0.47	0.15	0.14–0.16
	100	0.23	0.19–0.26	0.04	0.04–0.06

For peer review only

STROBE Statement—Checklist of items that should be included in reports of ***cohort studies***

	Item No	Recommendation	Page No
**Title and abstract**	1	(*a*) Indicate the study's design with a commonly used term in the title or the abstract	1
		(*b*) Provide in the abstract an informative and balanced summary of what was done and what was found	2-3
**Introduction**			
Background/rationale	2	Explain the scientific background and rationale for the investigation being reported	4-5
Objectives	3	State specific objectives, including any prespecified hypotheses	5
**Methods**			
Study design	4	Present key elements of study design early in the paper	6
Setting	5	Describe the setting, locations, and relevant dates, including periods of recruitment, exposure, follow-up, and data collection	6-7
Participants	6	(*a*) Give the eligibility criteria, and the sources and methods of selection of participants. Describe methods of follow-up	6
		(*b*) For matched studies, give matching criteria and number of exposed and unexposed	
Variables	7	Clearly define all outcomes, exposures, predictors, potential confounders, and effect modifiers. Give diagnostic criteria, if applicable	6-8
Data sources/ measurement	8*	For each variable of interest, give sources of data and details of methods of assessment (measurement). Describe comparability of assessment methods if there is more than one group	6-7
Bias	9	Describe any efforts to address potential sources of bias	8-9, 14-15
Study size	10	Explain how the study size was arrived at	6
Quantitative variables	11	Explain how quantitative variables were handled in the analyses. If applicable, describe which groupings were chosen and why	6-9
Statistical methods	12	(*a*) Describe all statistical methods, including those used to control for confounding	8-9
		(*b*) Describe any methods used to examine subgroups and interactions	9
		(*c*) Explain how missing data were addressed	6
		(*d*) If applicable, explain how loss to follow-up was addressed	8
		(*e*) Describe any sensitivity analyses	14-15
**Results**			
Participants	13*	(a) Report numbers of individuals at each stage of study—eg numbers potentially eligible, examined for eligibility, confirmed eligible, included in the study, completing follow-up, and analysed	9-10
		(b) Give reasons for non-participation at each stage	NA
		(c) Consider use of a flow diagram	
Descriptive data	14*	(a) Give characteristics of study participants (eg demographic, clinical, social) and information on exposures and potential confounders	9-10
		(b) Indicate number of participants with missing data for each variable of interest	6-7
		(c) Summarise follow-up time (eg, average and total amount)	10
Outcome data	15*	Report numbers of outcome events or summary measures over time	10

For peer review only

Main results	16	(*a*) Give unadjusted estimates and, if applicable, confounder-adjusted estimates and their precision (eg, 95% confidence interval). Make clear which confounders were adjusted for and why they were included<br>(*b*) Report category boundaries when continuous variables were categorized<br>(*c*) If relevant, consider translating estimates of relative risk into absolute risk for a meaningful time period	10-11
Other analyses	17	Report other analyses done—eg analyses of subgroups and interactions, and sensitivity analyses	9
**Discussion**			
Key results	18	Summarise key results with reference to study objectives	11
Limitations	19	Discuss limitations of the study, taking into account sources of potential bias or imprecision. Discuss both direction and magnitude of any potential bias	14-15
Interpretation	20	Give a cautious overall interpretation of results considering objectives, limitations, multiplicity of analyses, results from similar studies, and other relevant evidence	11-13
Generalisability	21	Discuss the generalisability (external validity) of the study results	14-15
**Other information**			
Funding	22	Give the source of funding and the role of the funders for the present study and, if applicable, for the original study on which the present article is based	19

*Give information separately for exposed and unexposed groups.

Note: An Explanation and Elaboration article discusses each checklist item and gives methodological background and published examples of transparent reporting. The STROBE checklist is best used in conjunction with this article (freely available on the Web sites of PLoS Medicine at http://www.plosmedicine.org/, Annals of Internal Medicine at http://www.annals.org/, and Epidemiology at http://www.epidem.com/). Information on the STROBE Initiative is available at http://www.strobe-statement.org.

For peer review only

For peer review only - http://bmjopen.bmj.com/site/about/guidelines.xhtml
